# Supplementary figures and images for: Model-Free Estimation of Tuning Curves and Their Attentional Modulation, Based on Sparse and Noisy Data
Source: PLoS One. 2016 Jan 19;11(1):e0146500. doi: 10.1371/journal.pone.0146500 (PMC4718600; doi:10.1371/journal.pone.0146500)

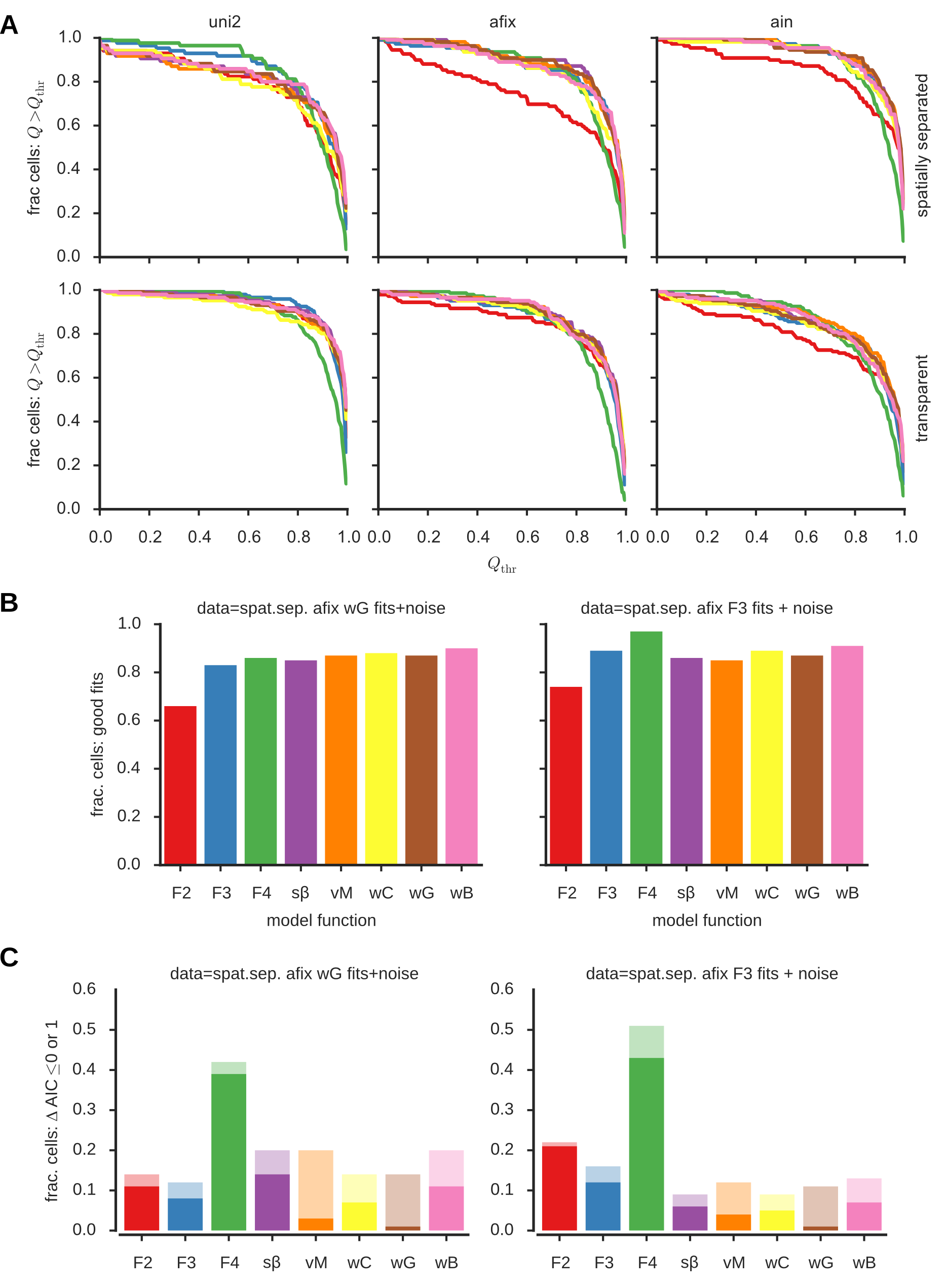

Supplement: S1 Fig — A) The threshold value for Q above which to accept a fit is not critical. Even for Qthr > 0.7 more than 80% of all cells lie above this threshold indicating a good fit. B) Model selection is ambiguous even for surrogate data based on an actual “ground-truth” model shape. We generated two surrogate ensembles of synthetic response data, by assuming that irregularity of shape derives just from noisy fluctuations added on top of underlying well-behaved tuning curves. We used as “true” shapes wG (left) or F3 (right) fits to the spatially separated afix condition and evaluated surrogate responses at the same angles as the measured tuning curves by adding noise to each of the 12 resulting points according to a Gaussian distribution with variance matched to empirical trial variability for each specific cell and stimulus direction. Despite the fact that a correct model now existed by construction, it was still impossible, for both ensembles, to infer it just based on quality of fit analyses. C) Computing the ΔAIC criterion for the surrogate data ensembles as in Fig 3C, the F4 fit always scored as the best, given its larger number of parameters. Nevertheless, among the five models with seven free parameters (F3, sβ, vM, wC and wG), F3 scored first when the underlying shape was, correctly, F3 (right), but not anymore when it was wrapped Gaussian (left), suggesting that superior performance of Fourier fits to empirical data reflects actual deviations from Gaussian shapes, rather than just better description of noise. (TIFF) [file pone.0146500.s001.tiff]

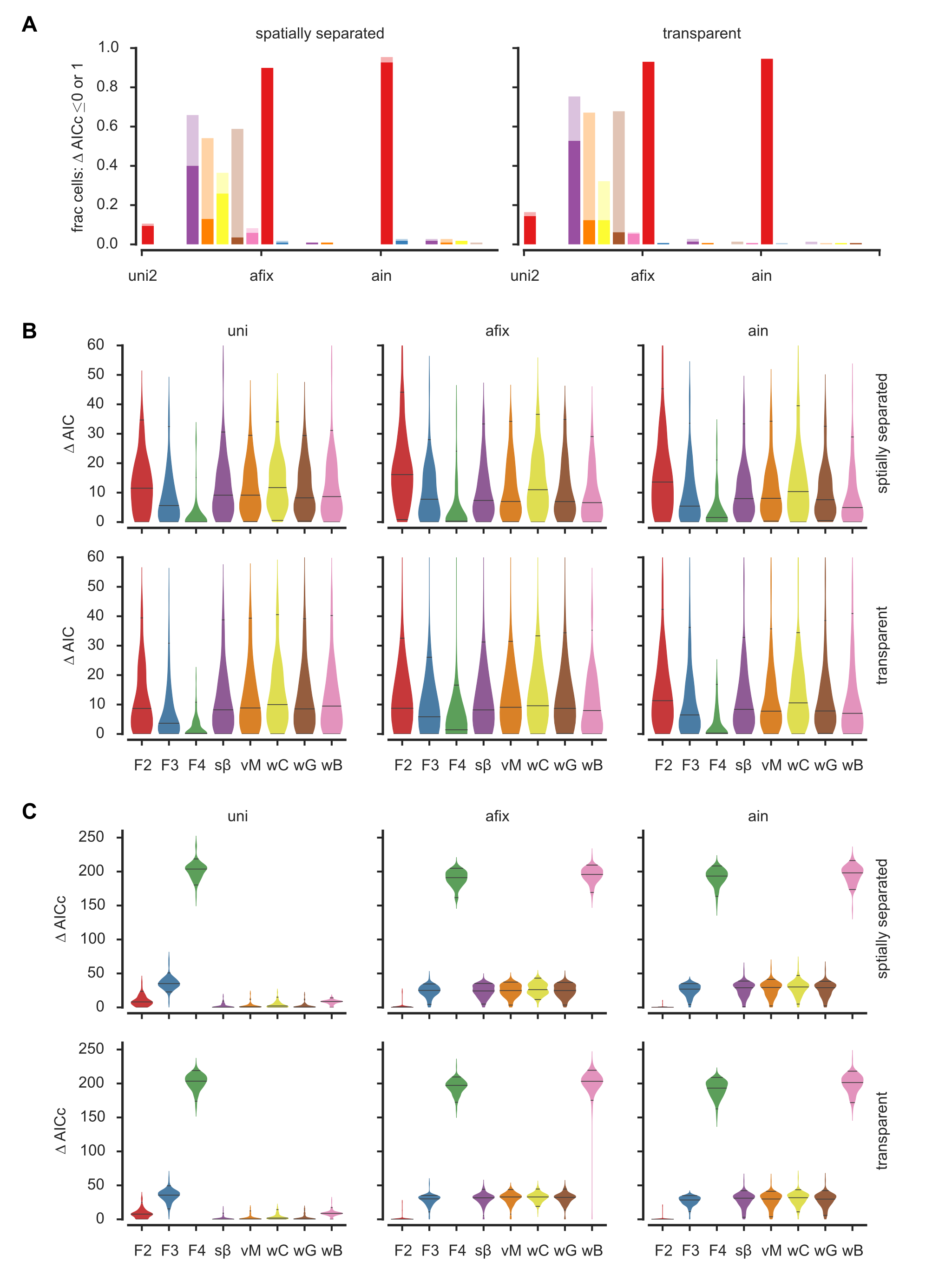

Supplement: S2 Fig — A) Layout as in Fig 3C, but showing ΔAICc instead of ΔAIC. Also for this criterion, none of the models is always selected, although for afix and ain conditions second order Fourier (F2) clearly performs best. B,C) Violinplots illustrating the distributions of ΔAIC (B) and ΔAICc (C). (TIFF) [file pone.0146500.s002.tiff]

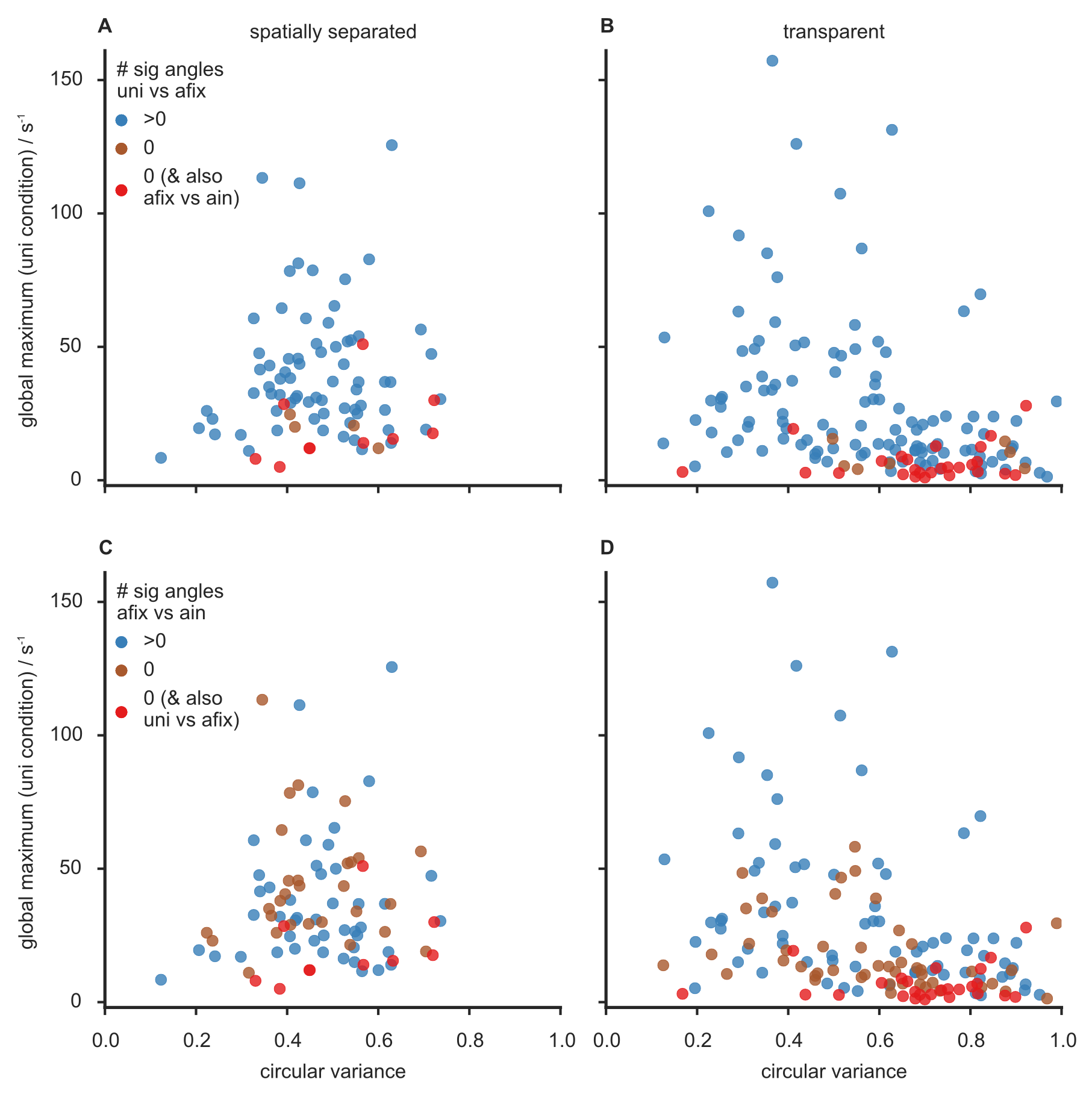

Supplement: S3 Fig — We compared trial ensembles for a given stimulus between conditions. Dots mark tuning properties of cells with at least one (blue) and zero (red, brown) significantly different stimuli between A,B) uni and afix condition, and C,D) afix and ain condition. Red dots mark cells without any significant change in both comparisons. These cells (A,B) tended to be badly tuned, but there were also equally badly tuned cells sensitive to this manipulation. Directing attention to the receptive field (C,D) had no clear relation to tuning properties. (TIFF) [file pone.0146500.s003.tiff]

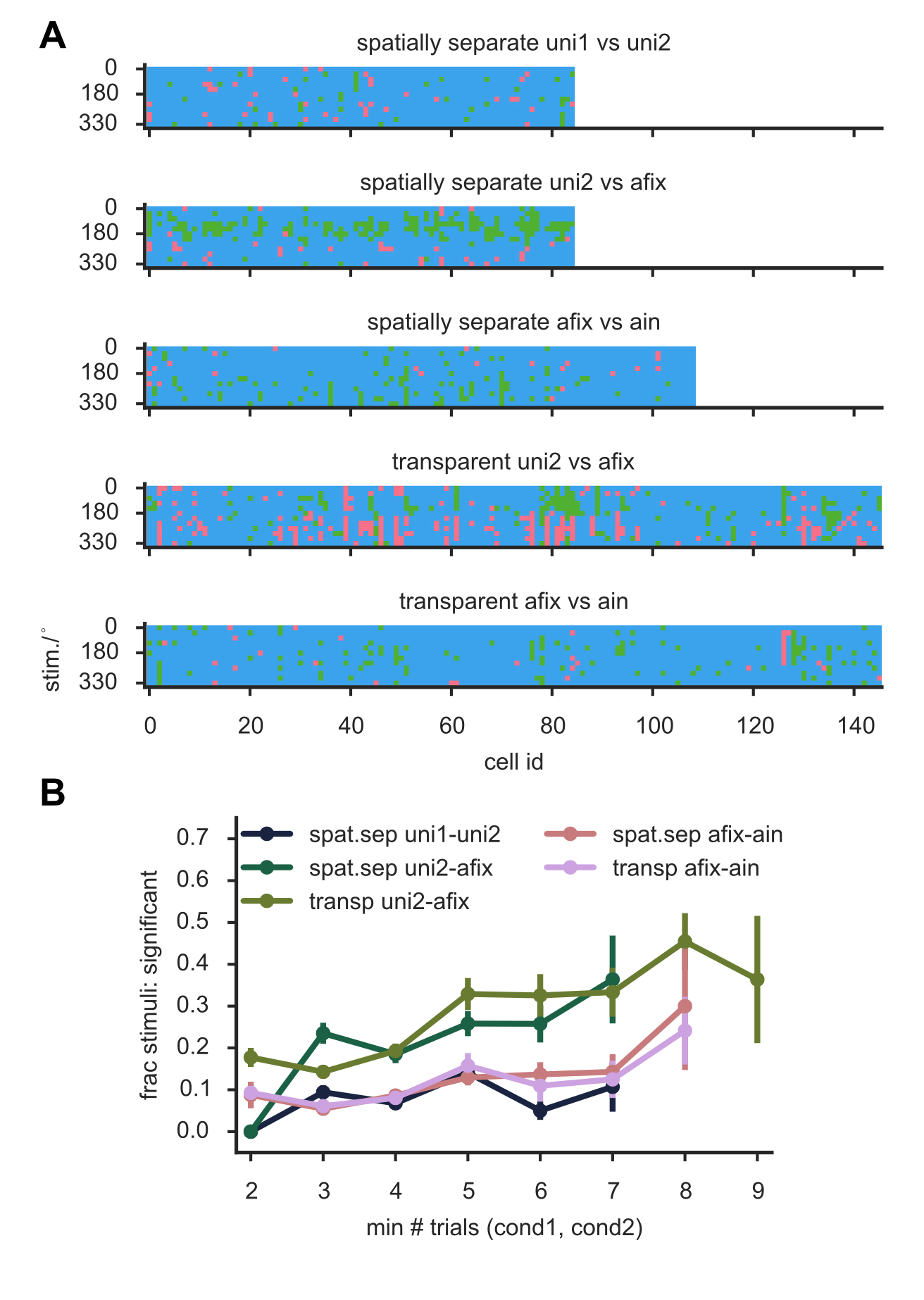

Supplement: S4 Fig — A) For each cell (x-axis) and stimulus (y-axis) colors indicate if there was a significant difference between the trials of the conditions marked in the title of each subplot. Colors are as in Fig 8. B) We determined the impact of the number of trials available for our various conditions on the number of cells exhibiting significant changes between conditions. The plot shows how the number of cells exhibiting significant changes between conditions varied as a function of the number of trials included in the analysis (using the smaller of the two ensemble sizes for the x-value). The fraction of significant changes for all the tested condition changes showed a clear trend to increase with the number of included trials, possibly saturating when the number of trials reached about 8. Error bars denote standard-error of the mean. Note that points are only shown in this plot when we had a minimum of 10 samples, on average we had between 170 and 250 samples (depending on condition). (TIFF) [file pone.0146500.s004.tiff]
