# Supplementary material for: Model-Free Estimation of Tuning Curves and Their Attentional Modulation, Based on Sparse and Noisy Data
Source: PLoS One. 2016 Jan 19;11(1):e0146500. doi: 10.1371/journal.pone.0146500 (PMC4718600; doi:10.1371/journal.pone.0146500)
Supplement: S1 Table — Each feature is calculated once for uni, once for afix and once for ain condition. (PDF) [file pone.0146500.s005.pdf]

Supporting Table S 1: List of features defined for all tuning curves. Each feature is calculated once for uni, once for afix and once for ain condition.

Feature name	Description
GlobalMinimumAngle [a]	$\operatorname{argmin} tc(\theta)$
GlobalMinimum	$\min tc(\theta)$
GlobalMaximumAngle	$\operatorname{argmax} tc(\theta)$
GlobalMaximum	$\max tc(\theta)$
PeakToPeak[b]	GlobalMaximum - GlobalMinimum
InnerMinimumAngle[c]	$\operatorname{argmin}_{\theta\in I} tc(\theta)$
InnerMinimum	tc(InnerMinimumAngle)
OuterMinimumAngle[d]	$\operatorname{argmin}_{\theta\in O} tc(\theta)$
MaximumAngle^{right} [e]	$\operatorname{argmax}_{\theta\in R} tc(\theta)$
InnerWidth^{right}	(MaximumAngle^{right} - InnerMinimumAngle) mod 360
OuterWidth^{right}	(OuterMinimumAngle - MaximumAngle^{right}) mod 360
Width^{right}	InnerWidth^{right} + OuterWidth^{right}
ΔWidth^{right}	OuterWidth^{right} - InnerWidth^{right}
Maximum^{right}	tc(MaximumAngle^{right})
PeakToPeak^{right}	tc(MaximumAngle^{right}) - GlobalMinimum
Skewness^{right} [f,g]	$\text{mom}_3(\tilde{R})/\text{mom}_2^{1.5}(\tilde{R})$
Kurtosis^{right}	$\text{mom}_4(\tilde{R})/\text{mom}_2^{2}(\tilde{R})$
Dip^{right}	Maximum^{right} - InnerMinimum
InnerBandwidth$^{\text{right}}_{X\,\%}$ [h]	bw(MaximumAngle^{right},left,X)
OuterBandwidth$^{\text{right}}_{X\,\%}$	bw(MaximumAngle^{right},right,X)
Bandwidth$^{\text{right}}_{X\,\%}$	InnerBandwidth$^{\text{right}}_{X\,\%}$ + OuterBandwidth$^{\text{right}}_{X\,\%}$
tcSymmetryIndex	$\sqrt{\sum_{\theta}(tc(\theta)-tc(360-\theta))^2/2}$

[a] The value of this circular variable was chosen to lie in the range $[-120°, 240°)$

[b] if PeakToPeak $< 10^{-10}$ don't calculate any feature at all

[c] $I = [0, 360)$ for uni conditions; for afix and ain first find local maxima for $45 \leq \theta \leq 315$ (to avoid spurious local maxima require that there's no higher point in the tuning curve within 15° on both sides of the local maxima). If there's no such local maximum $I = (120, 240)$, if there's one at 180° $I = (120, 240)$; if there's one at $\theta_m \neq 180$ $I = (\theta_m, 240)$ or $I = (120, \theta_m)$ depending on if θ_m lower or greater than 180°; if there are two $I = (\theta_0, \theta_{-1})$ where $0 \leq \theta_0$ and $\theta_{-1} \leq 360$ are the local maxima closest to 0° and 360°; if there are more than two: if all of them are $\leq$ or $\geq$ 180°also use $I = (\theta_0, \theta_{-1})$, otherwise there's at least one to the left and at least one to the right of 180°; pick the highest on either side; if, in addition, there is a local maximum at 180°, calculate it's distance d to the left and right peak according to $((x_{\text{left/right}} - x_{180})/360)^2 + ((y_{\text{left/right}} - y_{180})/\text{ptp})^2$; replace the peak which is closer according to the measure d if the peak at 180°is higher than it

[d] $O = [0, 360)$ for uni conditions; for afix and ain $O = [0, 360) \setminus I$

[e] $R = [180, 300]$ for uni conditions; for afix and ain $R =$ [InnerMinimumAngle, 315]; if tc(MaximumAngle^{right}) = GlobalMinimum then MaximumAngle^{right} (and likewise all dependent features) is not defined

[f] $\text{mom}_n(X) = \sum_{x\in X}(x - \bar{X})^n/n$ and $\bar{X}$ is the mean of set X

[g] if $0 \leq$ InnerMinimumAngle $<$ OuterMinimumAngle ≤ 360 then $\tilde{R}$ = [InnerMinimumAngle, OuterMinimumAngle], otherwise $\tilde{R} = [0, 360) \setminus$ [OuterMinimumAngle,InnerMinimumAngle]

[h] $bw(m, d, X)$ is defined as follows given the tuning curve tc: starting from position m it returns the first angle θ in direction $d \in \{\text{left}, \text{right}\}$ for which $tc(\theta) < \frac{X}{100}$PeakToPeak. Additionally, if an inner bandwidths (denoted "I") calculated this way is greater than the distance d of the corresponding peak to InnerMinimumAngle, the bandwidth is set to d
