# Supplementary material for: Model-Free Estimation of Tuning Curves and Their Attentional Modulation, Based on Sparse and Noisy Data
Source: PLoS One. 2016 Jan 19;11(1):e0146500. doi: 10.1371/journal.pone.0146500 (PMC4718600; doi:10.1371/journal.pone.0146500)
Supplement: S2 Table — Each feature is calculated only for uni condition. (PDF) [file pone.0146500.s006.pdf]

Supporting Table S 2: List of features defined only for uni condition. Each feature is calculated only for uni condition.

Feature name	Description
CircularVariance[a]	$1 - \left\| \sum_{\theta_n} \tilde{tc}(\theta_n) \mathrm{e}^{2\pi i \theta_n / 360} / \sum_{\theta_n} \tilde{tc}(\theta_n) \right\|$

[a] $\tilde{tc}(\theta) = tc(\theta) - \min tc(\theta)$
