# Supplementary material for: Model-Free Estimation of Tuning Curves and Their Attentional Modulation, Based on Sparse and Noisy Data
Source: PLoS One. 2016 Jan 19;11(1):e0146500. doi: 10.1371/journal.pone.0146500 (PMC4718600; doi:10.1371/journal.pone.0146500)
Supplement: S3 Table — Each features is calcualted once for afix and once for ain condition. (PDF) [file pone.0146500.s007.pdf]

Supporting Table S 3: List of features defined only for afix and ain condition. Each features is calcualted once for afix and once for ain condition.

Feature name	Description
MaximumAngle^{left}[a]	$\underset{\theta \in L}{\text{argmax}}\, tc(\theta)$
ΔMaximumAngle	MaximumAngle^{right} - MaximumAngle^{left}
OuterWidth^{left}	(MaximumAngle^{left} - OuterMinimumAngle) $\bmod 360$
InnerWidth^{left}	(InnerMinimumAngle - MaximumAngle^{left}) $\bmod 360$
Width^{left}	InnerWidth^{left} + OuterWidth^{left}
ΔWidth^{left}	OuterWidth^{left} - InnerWidth^{left}
ΔOuterWidth^{left}	OuterWidth^{right} - OuterWidth^{left}
ΔInnerWidth^{left}	InnerWidth^{right} - InnerWidth^{left}
Maximum^{left}	tc(MaximumAngle^{left})
PeakToPeak^{left}	Maximum^{left} - GlobalMinimum
ΔMaximum	Maximum^{right} - Maximum^{left}
minusSkewness^{left}[b]	-$\text{mom}_3(\tilde{L})/\text{mom}_2^{1.5}(\tilde{L})$
Kurtosis^{left}	$\text{mom}_4(\tilde{L})/\text{mom}_2^{2}(\tilde{L})$
ΔSkewness	Skewness^{right} - minusSkewness^{left}
ΔKurtosis	Kurtosis^{right} - Kurtosis^{left}
Dip	(Maximum^{left}+Maximum^{right})/2 - InnerMinimum
Dip^{left}	Maximum^{left} - InnerMinimum
OuterBandwidth$^{\text{left}}_{X\,\%}$	bw(MaximumAngle^{left},left,X)
InnerBandwidth$^{\text{left}}_{X\,\%}$	bw(MaximumAngle^{left},right,X)
Bandwidth$^{\text{left}}_{X\,\%}$	InnerBandwidth$^{\text{left}}_{X\,\%}$ + OuterBandwidth$^{\text{left}}_{X\,\%}$

[a] $L = [45, \text{InnerMinimumAngle}]$; if tc(MaximumAngle^{left}) = GlobalMinimum then MaximumAngle^{left} (and likewise all dependent features) is not defined

[b] if $0 \leq$ OuterMinimumAngle $<$ InnerMinimumAngle ≤ 360 then $\tilde{L}$ = [OuterMinimumAngle,InnerMinimumAngle], otherwise $\tilde{L} = [0, 360) \setminus$ [InnerMinimumAngle,OuteMinimumAngle]
