# Supplementary material for: Model-Free Estimation of Tuning Curves and Their Attentional Modulation, Based on Sparse and Noisy Data
Source: PLoS One. 2016 Jan 19;11(1):e0146500. doi: 10.1371/journal.pone.0146500 (PMC4718600; doi:10.1371/journal.pone.0146500)
Supplement: S4 Table — Conditions A,B can be either of uni, afix, ain. (PDF) [file pone.0146500.s008.pdf]

Supporting Table S 4: List of additional features comparing two conditions. Conditions A,B can be either of uni, afix, ain.

Feature name	Description
$\Delta\textsc{Maximum}^{\text{left}}_{\text{A,B}}$	$\textsc{Maximum}^{\text{left}}_{\text{B}}$ - $\textsc{Maximum}^{\text{left}}_{\text{A}}$
$\Delta\textsc{minusSkewness}^{\text{left}}_{\text{A,B}}$	$\textsc{minusSkewness}^{\text{left}}_{\text{B}}$ - $\textsc{minusSkewness}^{\text{left}}_{\text{A}}$
$\Delta\textsc{Kurtosis}^{\text{left}}_{\text{A,B}}$	$\textsc{Kurtosis}^{\text{left}}_{\text{B}}$ - $\textsc{Kurtosis}^{\text{left}}_{\text{A}}$
$\Delta\textsc{Maximum}^{\text{right}}_{\text{A,B}}$	$\textsc{Maximum}^{\text{right}}_{\text{B}}$ - $\textsc{Maximum}^{\text{right}}_{\text{A}}$
$\Delta\textsc{Skewness}^{\text{right}}_{\text{A,B}}$	$\textsc{Skewness}^{\text{right}}_{\text{B}}$ - $\textsc{Skewness}^{\text{right}}_{\text{A}}$
$\Delta\textsc{Kurtosis}^{\text{right}}_{\text{A,B}}$	$\textsc{Kurtosis}^{\text{right}}_{\text{B}}$ - $\textsc{Kurtosis}^{\text{right}}_{\text{A}}$
$\Delta\textsc{GlobalMinimum}_{\text{A,B}}$	$\textsc{GlobalMinimum}_{\text{B}}$ - $\textsc{GlobalMinimum}_{\text{A}}$
