# Supplementary material for: Model-Free Estimation of Tuning Curves and Their Attentional Modulation, Based on Sparse and Noisy Data
Source: PLoS One. 2016 Jan 19;11(1):e0146500. doi: 10.1371/journal.pone.0146500 (PMC4718600; doi:10.1371/journal.pone.0146500)
Supplement: S5 Table — The features in each row were compaired against each other within one condition (uni, afic or ain) and for all conditions they are defined. (PDF) [file pone.0146500.s009.pdf]

Supporting Table S 5: Feature pair categories. The features in each row were compaired against each other within one condition (uni, afic or ain) and for all conditions they are defined.

$\textsc{OuterWidth}^{\text{left}}$, $\textsc{OuterWidth}^{\text{right}}$
$\textsc{InnerWidth}^{\text{left}}$, $\textsc{InnerWidth}^{\text{right}}$
$\textsc{Width}^{\text{left}}$, $\textsc{Width}^{\text{right}}$
$\Delta\textsc{Width}^{\text{left}}$, $\Delta\textsc{Width}^{\text{right}}$
$\textsc{minusSkewness}^{\text{left}}$, $\textsc{Skewness}^{\text{right}}$
$\textsc{Kurtosis}^{\text{left}}$, $\textsc{Kurtosis}^{\text{right}}$
$\textsc{Maximum}^{\text{left}}$, $\textsc{Maximum}^{\text{right}}$
$\textsc{PeakToPeak}^{\text{left}}$, $\textsc{PeakToPeak}^{\text{right}}$
$\textsc{Dip}^{\text{left}}$, $\textsc{Dip}^{\text{right}}$
$\textsc{Bandwidth}^{\text{left}}_{75\,\%}$, $\textsc{Bandwidth}^{\text{right}}_{75\,\%}$
$\textsc{InnerBandwidth}^{\text{left}}_{75\,\%}$, $\textsc{InnerBandwidth}^{\text{right}}_{75\,\%}$, $\textsc{OuterBandwidth}^{\text{left}}_{75\,\%}$, $\textsc{OuterBandwidth}^{\text{right}}_{75\,\%}$
$\Delta\textsc{OuterWidth}$,$\Delta\textsc{InnerWidth}$
$\Delta\textsc{Maximum}^{\text{left}}$,$\Delta\textsc{Maximum}^{\text{right}}$
$\Delta\textsc{minusSkewness}^{\text{left}}$,$\Delta\textsc{Skewness}^{\text{right}}$
$\Delta\textsc{Kurtosis}^{\text{left}}$,$\Delta\textsc{Kurtosis}^{\text{right}}$
