# Supplementary material for: Model-Free Estimation of Tuning Curves and Their Attentional Modulation, Based on Sparse and Noisy Data
Source: PLoS One. 2016 Jan 19;11(1):e0146500. doi: 10.1371/journal.pone.0146500 (PMC4718600; doi:10.1371/journal.pone.0146500)
Supplement: S6 Table — Table lists cell count, mean, standard deviation, minimum, 25% quantile, median, 75% quantle and maximum for all features when evaluated with the direct method (values from best model in parentheses). (PDF) [file pone.0146500.s010.pdf]

Supporting Table S 6: Spatially separate paradigm's statistics for all features. Table lists cell count, mean, standard deviation, minimum, 25 % quantile, median, 75 % quantle and maximum for all features when evaluated with the direct method (values from best model in parentheses).

		count	mean	std	min	25%	50%	75%	max
afix	ΔInnerWidth	109	8.26 (4.31)	47.41 (43.80)	-90.00 (-133.90)	-30.00 (-12.50)	0.00 (8.70)	30.00 (24.60)	120.00 (155.90)
afix	ΔKurtosis	109	-0.15 (-0.06)	0.78 (0.99)	-2.69 (-3.61)	-0.61 (-0.35)	-0.11 (-0.06)	0.30 (0.15)	2.48 (3.85)
afix	ΔMaximum	109	2.98 (2.68)	13.98 (15.40)	-63.00 (-68.92)	-3.40 (-5.60)	3.60 (3.21)	10.67 (9.97)	38.80 (47.58)
afix	normalizedΔMaximum	85	0.03 (0.04)	0.41 (0.43)	-1.19 (-1.12)	-0.20 (-0.25)	0.08 (0.07)	0.27 (0.27)	0.98 (1.09)
afix	ΔOuterWidth	109	12.66 (22.18)	68.23 (58.93)	-180.00 (-124.10)	-30.00 (-15.90)	30.00 (32.60)	60.00 (62.60)	150.00 (126.60)
afix	ΔPeakToPeak	109	2.98 (2.68)	13.98 (15.40)	-63.00 (-68.92)	-3.40 (-5.60)	3.60 (3.21)	10.67 (9.97)	38.80 (47.58)
afix	normalizedΔPeakToPeak	85	0.03 (0.04)	0.41 (0.43)	-1.19 (-1.12)	-0.20 (-0.25)	0.08 (0.07)	0.27 (0.27)	0.98 (1.09)
afix	ΔSkewness	109	0.18 (-0.09)	0.92 (1.00)	-2.18 (-2.61)	-0.43 (-0.56)	0.11 (-0.09)	0.86 (0.41)	2.19 (2.95)
afix	ΔWidth	109	11.01 (23.18)	86.47 (84.19)	-180.00 (-166.40)	-60.00 (-23.00)	0.00 (35.60)	60.00 (78.00)	120.00 (177.80)
afix	Dip	109	14.41 (15.79)	9.20 (10.26)	2.47 (2.76)	8.75 (9.80)	12.50 (13.62)	17.30 (18.90)	56.79 (64.28)
afix	normalizedDip	85	0.42 (0.47)	0.22 (0.24)	0.05 (0.06)	0.28 (0.30)	0.39 (0.44)	0.52 (0.61)	1.47 (1.34)
afix	GlobalMaximum	109	43.41 (44.06)	30.97 (32.62)	4.00 (4.70)	22.00 (21.58)	34.50 (35.15)	55.43 (52.43)	167.50 (168.79)
afix	GlobalMaximumAngle	109	192.39 (192.80)	67.36 (65.92)	60.00 (50.30)	120.00 (124.50)	210.00 (222.90)	240.00 (246.30)	300.00 (306.40)
afix	normalizedGlobalMaximum	85	1.05 (1.10)	0.26 (0.26)	0.21 (0.25)	0.87 (0.92)	1.00 (1.08)	1.20 (1.26)	2.14 (1.97)
afix	GlobalMinimum	109	13.52 (13.40)	18.10 (18.71)	0.00 (-1.50)	2.67 (2.79)	7.14 (6.78)	15.67 (14.58)	101.50 (100.00)
afix	GlobalMinimumAngle	109	15.69 (21.42)	50.10 (56.15)	-60.00 (-64.40)	-30.00 (-15.20)	0.00 (12.80)	30.00 (41.60)	210.00 (218.90)
afix	normalizedGlobalMinimum	85	0.22 (0.22)	0.18 (0.18)	0.00 (-0.08)	0.07 (0.07)	0.20 (0.20)	0.32 (0.31)	0.81 (0.77)
afix	innerMinimumAngle	109	174.50 (175.73)	29.77 (30.29)	120.00 (69.60)	150.00 (157.50)	180.00 (174.00)	210.00 (196.20)	240.00 (250.40)
afix	innerMinimumVal	109	23.84 (22.79)	24.05 (23.58)	0.00 (-1.50)	8.80 (7.84)	16.00 (15.29)	29.50 (28.06)	137.20 (128.80)
afix	normalizedinnerMinimumVal	85	0.47 (0.46)	0.24 (0.25)	0.00 (-0.08)	0.30 (0.31)	0.49 (0.46)	0.64 (0.63)	1.09 (1.10)
afix	Bandwidth$^{\text{left}}_{75\,\%}$	109	82.57 (58.68)	27.74 (26.64)	30.00 (18.90)	60.00 (43.70)	90.00 (53.10)	90.00 (67.50)	180.00 (159.10)
afix	ΔWidth^{left}	109	52.84 (49.12)	54.30 (45.13)	-90.00 (-65.10)	0.00 (18.00)	60.00 (50.60)	90.00 (80.00)	180.00 (152.70)
afix	Dip^{left}	109	12.92 (14.46)	11.38 (12.89)	0.00 (0.00)	5.60 (5.71)	9.80 (11.25)	16.00 (18.30)	63.00 (68.97)
afix	normalizedDip^{left}	85	0.40 (0.45)	0.34 (0.35)	0.00 (0.00)	0.19 (0.20)	0.31 (0.38)	0.52 (0.65)	2.02 (1.82)
afix	InnerWidth^{left}	109	57.52 (58.82)	28.03 (27.58)	0.00 (0.00)	30.00 (43.80)	60.00 (56.30)	90.00 (68.30)	150.00 (156.90)
afix	InnerBandwidth$^{\text{left}}_{75\,\%}$	109	39.36 (27.89)	18.57 (16.50)	0.00 (0.00)	30.00 (19.80)	30.00 (25.50)	60.00 (33.90)	120.00 (121.40)
afix	Kurtosis^{left}	109	-0.93 (-0.92)	0.58 (0.72)	-1.76 (-1.90)	-1.34 (-1.34)	-1.02 (-1.11)	-0.65 (-0.81)	1.32 (2.31)
afix	Maximum^{left}	109	36.76 (37.25)	29.43 (30.37)	1.00 (0.99)	18.00 (16.71)	29.50 (27.77)	47.33 (46.06)	167.50 (168.79)
afix	MaximumAngle^{left}	109	116.97 (116.91)	30.26 (31.93)	60.00 (45.50)	90.00 (97.40)	120.00 (115.80)	120.00 (138.40)	180.00 (190.30)
afix	normalizedMaximum^{left}	85	0.88 (0.91)	0.37 (0.39)	0.12 (0.12)	0.63 (0.66)	0.85 (0.91)	1.16 (1.19)	2.14 (1.97)
afix	OuterWidth^{left}	109	110.37 (107.94)	40.18 (35.31)	30.00 (38.80)	90.00 (78.10)	120.00 (106.70)	150.00 (135.10)	210.00 (191.30)
afix	OuterBandwidth$^{\text{left}}_{75\,\%}$	109	43.21 (30.79)	22.15 (19.38)	30.00 (9.40)	30.00 (21.10)	30.00 (25.80)	60.00 (32.30)	120.00 (124.30)
afix	PeakToPeak^{left}	109	23.24 (23.85)	15.69 (16.42)	1.00 (0.95)	12.67 (12.70)	21.00 (20.89)	29.20 (31.54)	84.50 (85.28)
afix	normalizedPeakToPeak^{left}	85	0.65 (0.69)	0.34 (0.36)	0.12 (0.12)	0.43 (0.43)	0.60 (0.64)	0.83 (0.91)	2.14 (1.96)
afix	Width^{left}	109	167.89 (166.76)	43.03 (44.48)	60.00 (62.90)	150.00 (140.30)	150.00 (160.20)	210.00 (190.80)	270.00 (286.10)
afix	maxAngleDist	109	123.30 (121.94)	36.13 (32.83)	60.00 (37.40)	90.00 (100.50)	120.00 (117.90)	150.00 (140.90)	240.00 (214.30)
afix	minusLeftSkewness	109	-0.10 (0.11)	0.66 (0.66)	-1.69 (-1.58)	-0.58 (-0.35)	-0.11 (0.14)	0.38 (0.48)	1.51 (1.89)
afix	outerMinimumAngle	109	108.99 (147.68)	135.63 (147.99)	0.00 (0.50)	0.00 (21.70)	30.00 (56.20)	300.00 (325.00)	330.00 (359.70)
afix	PeakToPeak	109	29.89 (30.67)	17.09 (18.25)	4.00 (4.34)	16.80 (16.89)	26.80 (26.89)	36.33 (35.29)	87.67 (87.61)
afix	normalizedPeakToPeak	85	0.83 (0.88)	0.28 (0.28)	0.21 (0.23)	0.62 (0.71)	0.83 (0.84)	0.94 (1.00)	2.14 (1.96)
afix	Bandwidth$^{\text{right}}_{75\,\%}$	109	84.22 (58.21)	25.32 (21.02)	60.00 (15.20)	60.00 (45.20)	90.00 (54.80)	90.00 (68.80)	180.00 (129.70)
afix	ΔWidth^{right}	109	60.55 (67.00)	52.60 (39.41)	-120.00 (-62.90)	30.00 (48.00)	60.00 (68.00)	90.00 (91.50)	180.00 (156.60)
afix	Dip^{right}	109	15.90 (17.13)	11.73 (12.77)	0.00 (0.00)	9.00 (8.88)	13.50 (14.56)	20.00 (23.63)	58.86 (63.92)

Continued on next page

Table 6 – *Continued from previous page*

		count	mean	std	min	25%	50%	75%	max
afix	normalizedDip^{right}	85	0.44 (0.49)	0.27 (0.29)	0.00 (0.00)	0.24 (0.33)	0.42 (0.45)	0.56 (0.60)	1.28 (1.28)
afix	InnerWidth^{right}	109	65.78 (63.12)	31.48 (27.16)	0.00 (0.00)	30.00 (49.60)	60.00 (61.70)	90.00 (74.80)	150.00 (180.00)
afix	InnerBandwidth$^{right}_{75\,\%}$	109	39.36 (26.07)	18.57 (10.21)	0.00 (0.00)	30.00 (21.70)	30.00 (25.90)	60.00 (31.00)	90.00 (56.80)
afix	Kurtosis^{right}	109	-1.07 (-0.98)	0.55 (0.81)	-1.86 (-1.93)	-1.41 (-1.39)	-1.22 (-1.21)	-0.86 (-0.84)	1.78 (2.74)
afix	Maximum^{right}	109	39.73 (39.92)	29.57 (30.35)	3.00 (2.31)	19.33 (18.89)	29.60 (30.34)	48.00 (47.89)	152.67 (163.97)
afix	MaximumAngle^{right}	109	240.28 (238.85)	24.66 (25.48)	180.00 (158.90)	240.00 (223.40)	240.00 (240.20)	240.00 (251.40)	300.00 (306.40)
afix	normalizedMaximum^{right}	85	0.91 (0.95)	0.22 (0.24)	0.21 (0.23)	0.80 (0.82)	0.94 (0.97)	1.04 (1.10)	1.44 (1.42)
afix	OuterWidth^{right}	109	126.33 (130.12)	36.30 (32.07)	30.00 (53.60)	90.00 (109.90)	120.00 (133.90)	150.00 (151.50)	210.00 (196.80)
afix	OuterBandwidth$^{right}_{75\,\%}$	109	44.86 (32.14)	19.42 (15.44)	30.00 (8.50)	30.00 (22.90)	30.00 (28.80)	60.00 (37.30)	120.00 (103.30)
afix	PeakToPeak^{right}	109	26.22 (26.52)	16.19 (17.12)	3.00 (2.31)	14.33 (14.02)	22.90 (23.58)	34.50 (33.21)	87.67 (87.61)
afix	normalizedPeakToPeak^{right}	85	0.69 (0.73)	0.23 (0.26)	0.21 (0.19)	0.54 (0.56)	0.68 (0.74)	0.83 (0.88)	1.30 (1.31)
afix	Skewness^{right}	109	0.08 (0.02)	0.51 (0.56)	-1.86 (-1.64)	-0.21 (-0.21)	0.14 (0.06)	0.42 (0.36)	1.27 (1.46)
afix	Width^{right}	109	192.11 (193.24)	43.03 (44.48)	90.00 (73.90)	150.00 (169.20)	210.00 (199.80)	210.00 (219.70)	300.00 (297.10)
afix	tcSymmetryIndex	109	0.40 (6.86)	0.13 (2.20)	0.14 (2.10)	0.30 (5.54)	0.43 (7.08)	0.48 (8.33)	0.76 (12.45)
afix vs ain	ΔGlobalMinimum	109	1.95 (1.57)	5.26 (4.27)	-16.67 (-7.38)	-0.20 (-0.08)	1.14 (0.35)	3.36 (2.20)	30.25 (19.22)
afix vs ain	normalizedΔGlobalMinimum	85	0.03 (0.03)	0.09 (0.06)	-0.20 (-0.14)	-0.02 (0.00)	0.02 (0.02)	0.08 (0.08)	0.29 (0.17)
afix vs ain	ΔLeftKurtosis	109	-0.14 (-0.03)	0.73 (0.79)	-1.96 (-1.20)	-0.67 (-0.49)	-0.14 (-0.16)	0.28 (0.13)	2.59 (3.59)
afix vs ain	ΔLeftMaximum	109	-1.05 (-2.16)	8.95 (7.45)	-29.94 (-14.82)	-6.00 (-7.01)	-1.00 (-3.10)	2.75 (1.64)	28.90 (24.98)
afix vs ain	normalizedΔLeftMaximum	85	-0.08 (-0.14)	0.22 (0.25)	-0.60 (-0.76)	-0.21 (-0.24)	-0.05 (-0.17)	0.04 (0.01)	0.59 (0.41)
afix vs ain	ΔMinimumusLeftSkewness	109	0.08 (0.00)	0.76 (0.50)	-1.65 (-0.95)	-0.48 (-0.29)	0.10 (0.02)	0.47 (0.33)	2.43 (0.91)
afix vs ain	ΔRightKurtosis	109	0.00 (-0.03)	0.76 (0.48)	-2.87 (-1.93)	-0.30 (-0.20)	0.00 (0.01)	0.46 (0.21)	3.27 (1.02)
afix vs ain	ΔRightMaximum	109	6.37 (3.10)	9.95 (6.25)	-14.67 (-11.49)	0.07 (-0.57)	4.57 (3.05)	10.18 (6.25)	45.90 (23.82)
afix vs ain	normalizedΔRightMaximum	85	0.18 (0.09)	0.32 (0.22)	-0.50 (-0.38)	-0.04 (-0.04)	0.11 (0.07)	0.30 (0.21)	1.37 (0.62)
afix vs ain	ΔRightSkewness	109	-0.14 (-0.08)	0.60 (0.43)	-1.70 (-1.25)	-0.50 (-0.26)	-0.08 (-0.01)	0.12 (0.17)	1.87 (0.55)
ain	ΔInnerWidth	109	25.32 (32.40)	52.31 (51.63)	-150.00 (-140.40)	0.00 (7.70)	30.00 (23.80)	60.00 (60.30)	150.00 (175.40)
ain	ΔKurtosis	109	-0.00 (-0.13)	0.82 (0.81)	-2.21 (-3.71)	-0.38 (-0.29)	-0.07 (-0.01)	0.50 (0.24)	3.93 (1.66)
ain	ΔMaximum	109	10.40 (9.91)	13.66 (14.10)	-52.11 (-53.34)	1.67 (1.68)	9.13 (8.46)	16.87 (17.92)	44.07 (39.76)
ain	normalizedΔMaximum	85	0.29 (0.27)	0.42 (0.41)	-0.86 (-0.85)	0.04 (0.02)	0.26 (0.26)	0.47 (0.48)	1.57 (1.58)
ain	ΔOuterWidth	109	12.11 (19.35)	67.34 (59.14)	-150.00 (-146.40)	-30.00 (-18.60)	0.00 (21.30)	60.00 (65.10)	150.00 (140.40)
ain	ΔPeakToPeak	109	10.40 (9.91)	13.66 (14.10)	-52.11 (-53.34)	1.67 (1.68)	9.13 (8.46)	16.87 (17.92)	44.07 (39.76)
ain	normalizedΔPeakToPeak	85	0.29 (0.27)	0.42 (0.41)	-0.86 (-0.85)	0.04 (0.02)	0.26 (0.26)	0.47 (0.48)	1.57 (1.58)
ain	ΔSkewness	109	-0.04 (-0.21)	0.87 (0.92)	-1.72 (-1.90)	-0.63 (-0.89)	-0.13 (-0.36)	0.46 (0.25)	2.46 (2.89)
ain	ΔWidth	109	1.10 (31.94)	93.09 (91.09)	-180.00 (-179.00)	-60.00 (-28.80)	0.00 (49.40)	60.00 (91.80)	120.00 (178.40)
ain	Dip	109	13.23 (14.43)	8.66 (10.16)	1.10 (0.00)	7.41 (7.30)	11.20 (12.15)	16.26 (19.51)	59.83 (66.73)
ain	normalizedDip	85	0.39 (0.42)	0.23 (0.26)	0.04 (0.00)	0.23 (0.25)	0.35 (0.38)	0.47 (0.52)	1.37 (1.29)
ain	GlobalMaximum	109	47.58 (47.18)	34.15 (33.78)	2.00 (2.00)	24.36 (23.60)	38.50 (37.87)	59.20 (62.41)	198.57 (196.01)
ain	GlobalMaximumAngle	109	223.21 (221.85)	58.66 (54.92)	60.00 (62.10)	210.00 (217.20)	240.00 (236.90)	270.00 (254.00)	330.00 (299.70)
ain	normalizedGlobalMaximum	85	1.14 (1.16)	0.32 (0.30)	0.28 (0.32)	0.91 (0.95)	1.09 (1.15)	1.29 (1.30)	2.00 (2.08)
ain	GlobalMinimum	109	15.47 (15.47)	20.27 (20.41)	0.00 (-0.37)	4.00 (4.27)	7.71 (8.09)	17.43 (16.54)	123.25 (125.63)
ain	GlobalMinimumAngle	109	18.99 (24.03)	50.77 (52.10)	-60.00 (-102.50)	0.00 (-15.00)	0.00 (18.10)	60.00 (49.10)	210.00 (202.50)
ain	normalizedGlobalMinimum	85	0.26 (0.27)	0.20 (0.21)	0.00 (-0.02)	0.11 (0.11)	0.23 (0.23)	0.37 (0.36)	0.79 (0.81)
ain	innerMinimumAngle	109	171.19 (164.07)	27.78 (36.01)	120.00 (69.40)	150.00 (139.70)	150.00 (165.50)	180.00 (187.30)	240.00 (291.40)
ain	innerMinimumVal	109	27.68 (26.18)	26.15 (25.53)	0.00 (-0.18)	9.00 (7.44)	18.80 (17.35)	38.33 (36.55)	154.50 (153.15)
ain	normalizedinnerMinimumVal	85	0.56 (0.55)	0.30 (0.34)	0.00 (-0.01)	0.35 (0.28)	0.57 (0.51)	0.76 (0.78)	1.67 (1.63)
ain	Bandwidth$^{left}_{75\,\%}$	109	74.59 (57.65)	25.70 (39.98)	30.00 (8.00)	60.00 (36.40)	60.00 (50.50)	90.00 (69.10)	150.00 (303.30)

Continued on next page

Table 6 – *Continued from previous page*

		count	mean	std	min	25%	50%	75%	max
ain	ΔWidth^{left}	109	66.61 (63.67)	61.83 (53.81)	-90.00 (-45.30)	30.00 (22.40)	60.00 (60.30)	90.00 (91.80)	210.00 (254.10)
ain	Dip^{left}	109	8.03 (9.47)	8.80 (10.07)	0.00 (0.00)	2.67 (2.62)	5.96 (6.21)	11.09 (13.96)	53.45 (58.49)
ain	normalizedDip^{left}	85	0.24 (0.28)	0.24 (0.27)	0.00 (0.00)	0.08 (0.08)	0.19 (0.23)	0.31 (0.39)	1.40 (1.33)
ain	InnerWidth^{left}	109	48.17 (46.88)	31.89 (25.98)	0.00 (0.00)	30.00 (35.50)	30.00 (46.60)	60.00 (58.70)	180.00 (140.40)
ain	InnerBandwidth$^{\text{left}}_{75\,\%}$	109	33.30 (23.75)	17.95 (15.30)	0.00 (0.00)	30.00 (14.80)	30.00 (23.40)	30.00 (30.70)	90.00 (94.60)
ain	Kurtosis^{left}	109	-1.07 (-0.98)	0.58 (0.75)	-2.00 (-1.88)	-1.47 (-1.43)	-1.25 (-1.18)	-0.81 (-0.88)	0.83 (2.66)
ain	Maximum^{left}	109	35.71 (35.65)	30.17 (30.19)	2.00 (1.05)	15.75 (14.98)	27.11 (27.89)	44.00 (42.97)	154.50 (156.25)
ain	MaximumAngle^{left}	109	123.03 (117.20)	32.39 (36.84)	60.00 (45.00)	120.00 (97.20)	120.00 (120.10)	150.00 (137.40)	210.00 (281.00)
ain	normalizedMaximum^{left}	85	0.80 (0.83)	0.35 (0.38)	0.15 (0.05)	0.56 (0.56)	0.79 (0.84)	0.99 (1.11)	1.87 (1.79)
ain	OuterWidth^{left}	109	114.77 (110.55)	45.62 (47.74)	30.00 (30.30)	90.00 (81.20)	120.00 (103.40)	150.00 (135.00)	300.00 (329.20)
ain	OuterBandwidth$^{\text{left}}_{75\,\%}$	109	41.28 (33.90)	17.70 (37.14)	30.00 (8.00)	30.00 (20.90)	30.00 (26.90)	60.00 (34.50)	120.00 (283.70)
ain	PeakToPeak^{left}	109	20.24 (20.18)	15.39 (15.76)	1.33 (1.18)	9.33 (8.87)	17.14 (16.69)	26.58 (27.30)	71.78 (75.53)
ain	normalizedPeakToPeak^{left}	85	0.54 (0.57)	0.30 (0.34)	0.05 (0.06)	0.34 (0.32)	0.50 (0.52)	0.64 (0.68)	1.56 (1.61)
ain	Width^{left}	109	162.94 (157.42)	48.71 (54.88)	60.00 (57.20)	120.00 (129.00)	150.00 (150.60)	180.00 (183.60)	390.00 (404.30)
ain	maxAngleDist	109	121.65 (126.16)	37.60 (37.70)	30.00 (20.80)	90.00 (102.30)	120.00 (120.00)	150.00 (151.00)	210.00 (238.10)
ain	minusLeftSkewness	109	-0.02 (0.14)	0.58 (0.62)	-1.59 (-1.83)	-0.31 (-0.26)	0.01 (0.20)	0.40 (0.60)	1.53 (1.81)
ain	outerMinimumAngle	109	97.43 (138.76)	129.11 (144.23)	0.00 (1.70)	0.00 (25.70)	30.00 (50.30)	90.00 (323.70)	330.00 (359.60)
ain	PeakToPeak	109	32.11 (31.71)	18.90 (18.85)	2.00 (2.00)	17.71 (16.66)	29.33 (28.10)	40.80 (40.01)	86.29 (88.97)
ain	normalizedPeakToPeak	85	0.88 (0.89)	0.35 (0.34)	0.26 (0.30)	0.61 (0.63)	0.80 (0.82)	1.05 (1.07)	1.94 (1.96)
ain	Bandwidth$^{\text{right}}_{75\,\%}$	109	94.95 (72.73)	28.44 (27.88)	60.00 (18.20)	60.00 (54.80)	90.00 (70.20)	120.00 (86.40)	180.00 (169.50)
ain	ΔWidth^{right}	109	53.39 (50.62)	51.54 (49.85)	-90.00 (-120.10)	30.00 (16.90)	60.00 (58.10)	90.00 (85.90)	150.00 (147.40)
ain	Dip^{right}	109	18.43 (19.38)	12.89 (14.31)	0.00 (0.00)	9.56 (9.19)	15.45 (15.64)	26.07 (26.99)	66.20 (74.96)
ain	normalizedDip^{right}	85	0.53 (0.56)	0.37 (0.38)	0.00 (0.00)	0.27 (0.27)	0.45 (0.51)	0.70 (0.77)	1.65 (1.81)
ain	InnerWidth^{right}	109	73.49 (79.28)	32.53 (36.99)	0.00 (0.00)	60.00 (54.30)	90.00 (72.70)	90.00 (101.60)	150.00 (194.10)
ain	InnerBandwidth$^{\text{right}}_{75\,\%}$	109	46.24 (33.98)	22.56 (18.43)	0.00 (0.00)	30.00 (24.20)	30.00 (30.40)	60.00 (38.90)	120.00 (125.40)
ain	Kurtosis^{right}	109	-1.07 (-1.10)	0.58 (0.45)	-1.86 (-1.93)	-1.42 (-1.36)	-1.15 (-1.21)	-0.90 (-0.92)	2.49 (0.51)
ain	Maximum^{right}	109	46.10 (45.56)	34.12 (33.60)	1.00 (0.87)	22.00 (22.59)	36.29 (35.76)	58.80 (57.95)	198.57 (196.01)
ain	MaximumAngle^{right}	109	244.68 (243.35)	26.51 (26.44)	180.00 (179.80)	240.00 (229.50)	240.00 (242.50)	270.00 (255.40)	300.00 (301.80)
ain	normalizedMaximum^{right}	85	1.09 (1.11)	0.33 (0.32)	0.20 (0.17)	0.88 (0.93)	1.04 (1.07)	1.25 (1.29)	1.93 (2.08)
ain	OuterWidth^{right}	109	126.88 (129.90)	35.97 (30.82)	60.00 (63.40)	90.00 (108.70)	120.00 (133.40)	150.00 (148.70)	240.00 (228.80)
ain	OuterBandwidth$^{\text{right}}_{75\,\%}$	109	48.72 (38.75)	23.38 (20.91)	30.00 (9.00)	30.00 (26.50)	30.00 (32.80)	60.00 (44.20)	150.00 (128.10)
ain	PeakToPeak^{right}	109	30.64 (30.09)	18.67 (18.62)	1.00 (0.87)	15.92 (15.50)	26.60 (25.40)	40.14 (39.56)	86.29 (88.97)
ain	normalizedPeakToPeak^{right}	85	0.83 (0.84)	0.35 (0.36)	0.20 (0.17)	0.58 (0.60)	0.76 (0.81)	1.04 (1.03)	1.80 (1.96)
ain	Skewness^{right}	109	-0.06 (-0.07)	0.54 (0.53)	-1.17 (-1.36)	-0.35 (-0.40)	-0.04 (-0.12)	0.25 (0.25)	2.03 (1.42)
ain	Width^{right}	109	200.37 (209.18)	45.25 (46.40)	90.00 (90.50)	180.00 (179.00)	210.00 (210.50)	240.00 (239.70)	330.00 (327.20)
ain	tcSymmetryIndex	109	0.44 (7.58)	0.15 (2.86)	0.13 (2.13)	0.31 (5.19)	0.45 (8.12)	0.55 (9.63)	0.77 (14.38)
uni	CircularVariance	85	0.47 (0.46)	0.13 (0.15)	0.12 (0.06)	0.39 (0.40)	0.46 (0.47)	0.55 (0.56)	0.74 (0.78)
uni	GlobalMaximum	85	37.34 (36.73)	23.80 (24.07)	5.00 (6.00)	20.00 (19.69)	31.00 (30.62)	47.60 (45.89)	125.60 (127.83)
uni	GlobalMaximumAngle	85	240.00 (242.81)	0.00 (16.64)	240.00 (212.90)	240.00 (232.00)	240.00 (241.20)	240.00 (251.40)	240.00 (296.80)
uni	normalizedGlobalMaximum	85	1.00 (1.00)	0.00 (0.00)	1.00 (1.00)	1.00 (1.00)	1.00 (1.00)	1.00 (1.00)	1.00 (1.00)
uni	GlobalMinimum	85	7.36 (7.25)	9.09 (9.33)	0.00 (-0.89)	1.33 (1.09)	4.50 (4.14)	9.33 (8.54)	49.60 (47.06)
uni	GlobalMinimumAngle	85	64.59 (63.41)	51.86 (52.48)	-60.00 (-70.70)	30.00 (37.40)	60.00 (67.30)	90.00 (102.70)	150.00 (156.30)
uni	normalizedGlobalMinimum	85	0.17 (0.17)	0.13 (0.15)	0.00 (-0.09)	0.06 (0.04)	0.14 (0.13)	0.24 (0.26)	0.58 (0.62)
uni	PeakToPeak	85	29.98 (29.48)	17.72 (18.22)	5.00 (6.56)	17.50 (16.76)	25.50 (24.52)	38.80 (35.27)	95.83 (93.39)

Continued on next page

Table 6 – *Continued from previous page*

		count	mean	std	min	25%	50%	75%	max
uni	normalizedPeakToPeak	85	0.83 (0.83)	0.13 (0.15)	0.42 (0.38)	0.76 (0.74)	0.86 (0.87)	0.94 (0.96)	1.00 (1.09)
uni	$\text{Bandwidth}^{\text{right}}_{75\,\%}$	85	94.24 (69.70)	32.45 (27.70)	60.00 (17.30)	60.00 (52.10)	90.00 (61.60)	120.00 (81.90)	210.00 (160.80)
uni	$\Delta\text{Width}^{\text{right}}$	85	9.18 (1.20)	103.72 (96.44)	-240.00 (-213.80)	-60.00 (-63.20)	0.00 (0.00)	60.00 (75.20)	180.00 (198.40)
uni	$\text{InnerWidth}^{\text{right}}$	85	175.41 (179.40)	51.86 (48.22)	90.00 (80.80)	150.00 (142.40)	180.00 (180.00)	210.00 (211.60)	300.00 (286.90)
uni	$\text{InnerBandwidth}^{\text{right}}_{75\,\%}$	85	44.12 (34.48)	19.96 (15.17)	30.00 (8.60)	30.00 (25.40)	30.00 (30.20)	60.00 (37.50)	120.00 (94.80)
uni	$\text{Kurtosis}^{\text{right}}$	85	-0.52 (-0.48)	1.08 (1.42)	-1.79 (-1.92)	-1.21 (-1.16)	-0.74 (-0.75)	-0.09 (-0.32)	5.34 (6.66)
uni	$\text{Maximum}^{\text{right}}$	85	37.34 (36.73)	23.80 (24.07)	5.00 (6.00)	20.00 (19.69)	31.00 (30.62)	47.60 (45.89)	125.60 (127.83)
uni	$\text{MaximumAngle}^{\text{right}}$	85	240.00 (242.81)	0.00 (16.64)	240.00 (212.90)	240.00 (232.00)	240.00 (241.20)	240.00 (251.40)	240.00 (296.80)
uni	$\text{normalizedMaximum}^{\text{right}}$	85	1.00 (1.00)	0.00 (0.00)	1.00 (1.00)	1.00 (1.00)	1.00 (1.00)	1.00 (1.00)	1.00 (1.00)
uni	$\text{OuterWidth}^{\text{right}}$	85	184.59 (180.60)	51.86 (48.22)	60.00 (73.10)	150.00 (148.40)	180.00 (180.00)	210.00 (217.60)	270.00 (279.20)
uni	$\text{OuterBandwidth}^{\text{right}}_{75\,\%}$	85	50.12 (35.21)	25.52 (16.55)	30.00 (8.70)	30.00 (25.70)	30.00 (31.00)	60.00 (39.10)	150.00 (98.50)
uni	$\text{PeakToPeak}^{\text{right}}$	85	29.98 (29.48)	17.72 (18.22)	5.00 (6.56)	17.50 (16.76)	25.50 (24.52)	38.80 (35.27)	95.83 (93.39)
uni	$\text{normalizedPeakToPeak}^{\text{right}}$	85	0.83 (0.83)	0.13 (0.15)	0.42 (0.38)	0.76 (0.74)	0.86 (0.87)	0.94 (0.96)	1.00 (1.09)
uni	$\text{Skewness}^{\text{right}}$	85	0.69 (0.71)	0.53 (0.58)	-0.41 (-0.37)	0.31 (0.33)	0.63 (0.64)	1.04 (1.00)	2.62 (2.80)
uni	$\text{Width}^{\text{right}}$	85	360.00 (360.00)	0.00 (0.00)	360.00 (360.00)	360.00 (360.00)	360.00 (360.00)	360.00 (360.00)	360.00 (360.00)
uni vs afix	ΔGlobalMinimum	85	3.30 (1.69)	7.93 (2.76)	-6.80 (-2.26)	-0.17 (-0.23)	1.60 (0.98)	4.17 (3.03)	58.00 (9.68)
uni vs afix	normalizedΔGlobalMinimum	85	0.06 (0.05)	0.10 (0.09)	-0.16 (-0.13)	-0.01 (-0.02)	0.05 (0.03)	0.11 (0.13)	0.46 (0.23)
uni vs afix	ΔRightMaximum	85	-2.40 (0.35)	7.22 (4.23)	-30.40 (-8.97)	-5.33 (-2.09)	-2.00 (-0.24)	0.67 (2.22)	15.90 (8.60)
uni vs afix	normalizedΔRightMaximum	85	-0.09 (-0.00)	0.22 (0.15)	-0.79 (-0.34)	-0.20 (-0.07)	-0.06 (-0.01)	0.04 (0.09)	0.44 (0.37)
uni vs ain	ΔGlobalMinimum	85	4.23 (3.62)	8.18 (5.52)	-6.27 (-5.26)	0.00 (0.36)	2.13 (2.88)	4.76 (4.91)	55.75 (16.89)
uni vs ain	normalizedΔGlobalMinimum	85	0.09 (0.09)	0.12 (0.13)	-0.14 (-0.19)	0.00 (0.01)	0.06 (0.07)	0.16 (0.15)	0.44 (0.33)
uni vs ain	ΔRightMaximum	85	2.95 (3.41)	11.55 (12.80)	-29.00 (-33.73)	-4.00 (-2.17)	1.25 (1.87)	7.56 (7.67)	44.40 (42.74)
uni vs ain	normalizedΔRightMaximum	85	0.09 (0.13)	0.33 (0.27)	-0.80 (-0.54)	-0.12 (-0.05)	0.04 (0.13)	0.25 (0.29)	0.93 (0.84)
