# Supplementary material for: Model-Free Estimation of Tuning Curves and Their Attentional Modulation, Based on Sparse and Noisy Data
Source: PLoS One. 2016 Jan 19;11(1):e0146500. doi: 10.1371/journal.pone.0146500 (PMC4718600; doi:10.1371/journal.pone.0146500)
Supplement: S7 Table — Table lists cell count, mean, standard deviation, minimum, 25% quantile, median, 75% quantle and maximum for all features when evaluated with the direct method (values from best model in parentheses). (PDF) [file pone.0146500.s011.pdf]

Supporting Table S 7: Transparent paradigm's statistics for all features. Table lists cell count, mean, standard deviation, minimum, 25 % quantile, median, 75 % quantle and maximum for all features when evaluated with the direct method (values from best model in parentheses).

		count	mean	std	min	25%	50%	75%	max
afix	ΔInnerWidth	143	0.63 (4.89)	53.34 (56.36)	-120.00 (-175.60)	-30.00 (-22.05)	0.00 (7.00)	30.00 (34.70)	150.00 (154.90)
afix	ΔKurtosis	144	-0.12 (0.07)	1.11 (1.15)	-2.85 (-4.26)	-0.77 (-0.42)	-0.09 (-0.01)	0.52 (0.45)	3.00 (4.90)
afix	ΔMaximum	143	0.97 (1.58)	7.53 (8.43)	-21.00 (-25.79)	-2.50 (-1.77)	0.00 (0.11)	3.33 (3.77)	43.00 (53.44)
afix	normalizedΔMaximum	143	0.02 (-0.16)	0.57 (2.29)	-2.00 (-26.40)	-0.19 (-0.13)	0.00 (0.02)	0.18 (0.22)	4.18 (2.82)
afix	ΔOuterWidth	143	-0.63 (7.33)	89.96 (81.96)	-180.00 (-165.60)	-60.00 (-51.80)	0.00 (2.10)	60.00 (72.80)	150.00 (178.50)
afix	ΔPeakToPeak	143	0.97 (1.58)	7.53 (8.43)	-21.00 (-25.79)	-2.50 (-1.77)	0.00 (0.11)	3.33 (3.77)	43.00 (53.44)
afix	normalizedΔPeakToPeak	143	0.02 (-0.16)	0.57 (2.29)	-2.00 (-26.40)	-0.19 (-0.13)	0.00 (0.02)	0.18 (0.22)	4.18 (2.82)
afix	ΔSkewness	144	0.51 (-0.09)	1.04 (1.20)	-2.22 (-2.99)	-0.15 (-0.79)	0.58 (-0.10)	1.19 (0.56)	3.58 (5.22)
afix	ΔWidth	143	-2.52 (-0.37)	95.77 (105.53)	-180.00 (-180.00)	-60.00 (-87.40)	0.00 (1.60)	60.00 (82.50)	120.00 (178.60)
afix	Dip	143	8.12 (8.15)	6.39 (6.90)	0.38 (0.39)	3.50 (3.21)	6.50 (6.52)	10.83 (10.93)	36.00 (38.09)
afix	normalizedDip	143	0.56 (0.72)	0.54 (1.26)	0.04 (0.03)	0.25 (0.23)	0.43 (0.45)	0.73 (0.77)	4.50 (13.20)
afix	GlobalMaximum	145	21.15 (20.66)	21.92 (22.34)	0.50 (0.76)	7.50 (6.39)	14.00 (12.94)	27.71 (26.66)	127.00 (130.39)
afix	GlobalMaximumAngle	145	173.59 (184.15)	86.76 (81.34)	0.00 (4.90)	120.00 (119.70)	180.00 (184.70)	240.00 (248.20)	330.00 (349.90)
afix	normalizedGlobalMaximum	145	1.14 (1.69)	1.10 (5.96)	0.07 (0.17)	0.62 (0.63)	0.93 (0.89)	1.43 (1.37)	11.57 (70.99)
afix	GlobalMinimum	145	5.01 (5.08)	8.56 (8.99)	0.00 (-1.38)	0.00 (0.27)	2.00 (1.53)	5.00 (5.50)	48.00 (51.41)
afix	GlobalMinimumAngle	145	38.90 (42.14)	78.11 (88.81)	-120.00 (-116.70)	0.00 (-15.75)	30.00 (26.40)	90.00 (89.35)	210.00 (238.50)
afix	normalizedGlobalMinimum	145	0.17 (0.40)	0.22 (2.57)	0.00 (-1.37)	0.00 (0.03)	0.12 (0.12)	0.28 (0.27)	2.00 (30.49)
afix	innerMinimumAngle	145	177.72 (179.87)	31.24 (34.74)	120.00 (108.20)	150.00 (154.40)	180.00 (180.60)	210.00 (203.50)	240.00 (269.50)
afix	innerMinimumVal	145	10.48 (9.94)	16.66 (16.20)	0.00 (-0.45)	1.33 (1.10)	4.00 (4.12)	12.00 (11.07)	99.43 (96.17)
afix	normalizedinnerMinimumVal	145	0.37 (0.69)	0.36 (3.73)	0.00 (-0.24)	0.15 (0.12)	0.32 (0.32)	0.51 (0.56)	3.27 (44.59)
afix	Bandwidth$^{left}_{75\%}$	144	76.88 (57.43)	25.51 (31.29)	30.00 (12.80)	60.00 (37.00)	60.00 (47.90)	90.00 (69.15)	180.00 (217.90)
afix	ΔWidth^{left}	144	59.58 (51.84)	79.09 (64.23)	-120.00 (-153.50)	0.00 (6.75)	60.00 (52.70)	120.00 (97.55)	240.00 (187.00)
afix	Dip^{left}	144	7.59 (7.36)	6.38 (6.63)	0.00 (0.00)	2.99 (2.44)	5.83 (5.10)	10.56 (10.53)	34.00 (35.40)
afix	normalizedDip^{left}	144	0.55 (0.80)	0.53 (2.31)	0.00 (0.00)	0.19 (0.16)	0.41 (0.43)	0.70 (0.73)	3.22 (26.40)
afix	InnerWidth^{left}	144	63.33 (66.69)	36.72 (36.63)	0.00 (0.00)	30.00 (45.70)	60.00 (57.10)	90.00 (87.30)	150.00 (212.10)
afix	InnerBandwidth$^{left}_{75\%}$	144	36.04 (25.20)	16.09 (14.00)	0.00 (0.00)	30.00 (16.80)	30.00 (22.40)	30.00 (31.70)	90.00 (89.10)
afix	Kurtosis^{left}	144	-0.79 (-0.82)	0.80 (0.94)	-3.00 (-2.00)	-1.34 (-1.34)	-0.95 (-1.00)	-0.47 (-0.66)	2.17 (6.26)
afix	Maximum^{left}	144	18.14 (17.30)	18.85 (18.78)	0.25 (0.43)	5.88 (4.98)	11.00 (10.37)	25.08 (23.79)	102.00 (108.84)
afix	MaximumAngle^{left}	144	114.58 (113.18)	36.29 (37.60)	60.00 (45.00)	90.00 (84.10)	120.00 (113.70)	150.00 (145.05)	210.00 (190.70)
afix	normalizedMaximum^{left}	144	0.92 (1.49)	0.67 (5.97)	0.12 (0.11)	0.49 (0.52)	0.76 (0.75)	1.04 (1.06)	4.67 (70.99)
afix	OuterWidth^{left}	144	122.92 (118.53)	65.83 (57.63)	30.00 (39.40)	90.00 (70.05)	120.00 (114.40)	150.00 (150.85)	330.00 (313.20)
afix	OuterBandwidth$^{left}_{75\%}$	144	40.83 (32.24)	21.17 (25.55)	30.00 (0.10)	30.00 (18.65)	30.00 (23.60)	60.00 (36.60)	150.00 (187.30)
afix	PeakToPeak^{left}	144	13.09 (12.22)	12.19 (11.88)	0.25 (0.00)	4.60 (4.24)	9.08 (9.12)	18.18 (16.64)	76.00 (75.32)
afix	normalizedPeakToPeak^{left}	144	0.74 (1.09)	0.59 (3.45)	0.12 (0.00)	0.36 (0.35)	0.51 (0.53)	0.95 (0.85)	3.41 (40.50)
afix	Width^{left}	144	186.25 (185.22)	71.48 (72.11)	60.00 (53.90)	150.00 (136.10)	180.00 (179.20)	210.00 (224.50)	480.00 (469.10)
afix	maxAngleDist	143	127.76 (138.27)	46.38 (46.73)	60.00 (61.50)	90.00 (100.35)	120.00 (129.40)	150.00 (170.75)	240.00 (270.00)
afix	minusLeftSkewness	144	-0.26 (0.06)	0.70 (0.70)	-2.04 (-2.73)	-0.69 (-0.36)	-0.28 (0.04)	0.19 (0.59)	1.49 (1.90)
afix	outerMinimumAngle	145	108.41 (168.36)	124.41 (140.15)	0.00 (1.90)	0.00 (33.25)	30.00 (92.10)	240.00 (318.15)	330.00 (359.40)
afix	PeakToPeak	145	16.14 (15.58)	14.96 (15.06)	0.50 (0.77)	6.00 (5.66)	11.93 (10.43)	20.00 (20.63)	86.50 (87.22)
afix	normalizedPeakToPeak	145	0.97 (1.29)	1.07 (3.45)	0.07 (0.17)	0.44 (0.48)	0.74 (0.72)	1.14 (1.11)	11.57 (40.50)
afix	Bandwidth$^{right}_{75\%}$	144	79.17 (59.65)	28.07 (33.27)	30.00 (0.40)	60.00 (37.90)	60.00 (50.00)	90.00 (76.45)	180.00 (195.20)
afix	ΔWidth^{right}	144	68.54 (46.73)	72.16 (64.02)	-90.00 (-145.70)	22.50 (1.20)	60.00 (45.70)	120.00 (89.25)	240.00 (223.00)
afix	Dip^{right}	143	8.60 (8.94)	8.34 (9.31)	0.00 (0.00)	3.00 (2.92)	6.07 (6.59)	11.13 (10.78)	46.67 (55.35)

Continued on next page

Table 7 – *Continued from previous page*

		count	mean	std	min	25%	50%	75%	max
afix	normalizedDip^{right}	143	0.57 (0.64)	0.69 (0.69)	0.00 (0.00)	0.22 (0.21)	0.41 (0.47)	0.72 (0.82)	6.59 (4.80)
afix	InnerWidth^{right}	144	63.96 (71.58)	33.89 (36.58)	0.00 (0.00)	30.00 (49.00)	60.00 (61.60)	90.00 (86.45)	180.00 (204.00)
afix	InnerBandwidth$^{right}_{75\%}$	144	35.83 (27.69)	14.75 (19.46)	0.00 (0.00)	30.00 (16.85)	30.00 (23.00)	30.00 (33.25)	90.00 (144.60)
afix	Kurtosis^{right}	145	-0.89 (-0.74)	0.74 (1.00)	-2.00 (-1.72)	-1.44 (-1.34)	-1.09 (-0.98)	-0.62 (-0.64)	1.67 (5.64)
afix	Maximum^{right}	144	19.10 (18.88)	21.80 (22.16)	0.50 (0.03)	6.00 (5.11)	12.00 (11.57)	26.00 (24.68)	127.00 (130.39)
afix	MaximumAngle^{right}	144	241.67 (251.45)	33.62 (35.63)	150.00 (168.50)	210.00 (229.30)	240.00 (247.50)	270.00 (272.55)	300.00 (315.00)
afix	normalizedMaximum^{right}	144	0.94 (1.33)	0.76 (3.78)	0.07 (0.01)	0.53 (0.58)	0.78 (0.76)	1.12 (1.22)	7.07 (44.59)
afix	OuterWidth^{right}	144	132.50 (118.31)	59.31 (53.97)	30.00 (12.80)	90.00 (69.90)	120.00 (118.00)	180.00 (148.50)	330.00 (313.50)
afix	OuterBandwidth$^{right}_{75\%}$	144	43.33 (31.96)	22.93 (23.33)	30.00 (0.40)	30.00 (18.95)	30.00 (25.60)	60.00 (35.80)	150.00 (150.00)
afix	PeakToPeak^{right}	144	14.05 (13.79)	14.83 (15.05)	0.50 (0.00)	4.69 (4.18)	9.50 (8.70)	17.62 (16.57)	86.50 (87.22)
afix	normalizedPeakToPeak^{right}	144	0.76 (0.93)	0.73 (1.34)	0.07 (0.00)	0.41 (0.40)	0.55 (0.62)	0.96 (1.05)	7.07 (14.11)
afix	Skewness^{right}	145	0.26 (-0.03)	0.64 (0.74)	-1.21 (-2.14)	-0.21 (-0.55)	0.24 (-0.05)	0.66 (0.44)	1.79 (2.49)
afix	Width^{right}	144	196.46 (189.89)	64.24 (66.37)	60.00 (12.80)	150.00 (144.80)	195.00 (187.80)	240.00 (237.20)	450.00 (451.80)
afix	tcSymmetryIndex	145	0.40 (6.96)	0.13 (2.41)	0.00 (0.38)	0.31 (5.29)	0.41 (6.71)	0.49 (8.73)	0.72 (12.73)
afix vs ain	ΔGlobalMinimum	145	1.07 (1.78)	6.41 (4.10)	-30.00 (-2.86)	-0.90 (-0.34)	0.13 (0.38)	1.53 (2.28)	48.57 (15.96)
afix vs ain	normalizedΔGlobalMinimum	145	0.03 (0.08)	0.24 (0.14)	-2.00 (-0.10)	-0.04 (-0.02)	0.01 (0.05)	0.13 (0.13)	0.77 (0.52)
afix vs ain	ΔLeftKurtosis	143	-0.10 (0.13)	1.07 (0.93)	-3.71 (-3.97)	-0.76 (-0.26)	-0.05 (0.04)	0.53 (0.60)	2.52 (1.63)
afix vs ain	ΔLeftMaximum	143	4.35 (8.18)	14.29 (16.93)	-63.00 (-10.86)	-1.08 (-0.31)	1.70 (3.11)	6.34 (9.36)	88.50 (76.93)
afix vs ain	normalizedΔLeftMaximum	143	0.08 (0.17)	0.60 (0.46)	-4.20 (-1.27)	-0.12 (-0.04)	0.13 (0.20)	0.31 (0.36)	2.40 (1.27)
afix vs ain	ΔMinimumusLeftSkewness	143	0.06 (0.06)	0.84 (0.69)	-1.97 (-1.65)	-0.53 (-0.27)	0.14 (0.05)	0.60 (0.36)	2.21 (2.51)
afix vs ain	ΔRightKurtosis	145	0.02 (0.14)	1.03 (1.04)	-2.65 (-1.75)	-0.49 (-0.61)	0.01 (0.16)	0.54 (0.71)	3.30 (3.46)
afix vs ain	ΔRightMaximum	144	4.65 (7.10)	13.12 (15.52)	-38.00 (-6.69)	-1.08 (0.28)	1.65 (3.22)	7.86 (7.23)	78.83 (87.33)
afix vs ain	normalizedΔRightMaximum	144	0.14 (0.18)	0.81 (0.47)	-5.38 (-1.06)	-0.11 (0.04)	0.13 (0.14)	0.44 (0.37)	4.17 (2.05)
afix vs ain	ΔRightSkewness	145	-0.03 (-0.01)	0.82 (0.84)	-1.75 (-1.93)	-0.59 (-0.51)	-0.07 (-0.12)	0.54 (0.38)	2.14 (1.81)
ain	ΔInnerWidth	145	9.93 (-1.12)	53.74 (50.55)	-150.00 (-135.40)	-30.00 (-26.90)	0.00 (3.45)	30.00 (23.32)	150.00 (136.40)
ain	ΔKurtosis	145	0.01 (0.03)	1.06 (1.01)	-3.52 (-3.32)	-0.54 (-0.46)	-0.10 (-0.03)	0.53 (0.44)	4.21 (4.16)
ain	ΔMaximum	145	1.29 (1.18)	12.84 (12.98)	-67.20 (-39.83)	-2.45 (-3.38)	0.50 (0.40)	4.54 (3.38)	91.50 (104.98)
ain	normalizedΔMaximum	145	0.08 (0.14)	0.66 (0.99)	-3.40 (-4.07)	-0.20 (-0.19)	0.05 (0.05)	0.29 (0.27)	3.45 (6.52)
ain	ΔOuterWidth	145	-6.21 (7.12)	92.19 (81.85)	-180.00 (-170.10)	-60.00 (-50.72)	0.00 (4.65)	60.00 (63.00)	150.00 (178.20)
ain	ΔPeakToPeak	145	1.29 (1.18)	12.84 (12.98)	-67.20 (-39.83)	-2.45 (-3.38)	0.50 (0.40)	4.54 (3.38)	91.50 (104.98)
ain	normalizedΔPeakToPeak	145	0.08 (0.14)	0.66 (0.99)	-3.40 (-4.07)	-0.20 (-0.19)	0.05 (0.05)	0.29 (0.27)	3.45 (6.52)
ain	ΔSkewness	145	0.43 (-0.03)	0.95 (1.08)	-2.27 (-2.51)	-0.27 (-0.74)	0.42 (0.00)	1.02 (0.58)	3.14 (3.12)
ain	ΔWidth	145	-8.69 (-3.86)	96.43 (97.31)	-180.00 (-178.60)	-60.00 (-79.30)	0.00 (1.10)	60.00 (55.90)	120.00 (177.40)
ain	Dip	145	9.95 (10.46)	10.17 (10.54)	0.30 (0.02)	4.07 (3.67)	6.90 (7.01)	11.55 (13.12)	64.40 (57.75)
ain	normalizedDip	145	0.59 (0.87)	0.46 (2.52)	0.06 (0.02)	0.27 (0.33)	0.48 (0.57)	0.73 (0.80)	3.18 (30.32)
ain	GlobalMaximum	146	26.52 (26.18)	29.44 (29.83)	1.60 (0.45)	8.00 (6.38)	15.75 (15.93)	33.69 (33.30)	150.57 (151.46)
ain	GlobalMaximumAngle	146	180.00 (189.61)	80.73 (79.79)	0.00 (1.10)	120.00 (120.70)	180.00 (206.35)	240.00 (250.30)	330.00 (356.00)
ain	normalizedGlobalMaximum	146	1.25 (1.53)	0.71 (2.66)	0.31 (0.22)	0.83 (0.80)	1.08 (1.14)	1.44 (1.53)	4.80 (31.50)
ain	GlobalMinimum	146	6.04 (5.84)	11.48 (11.72)	0.00 (-3.49)	0.41 (0.20)	1.93 (1.44)	6.09 (5.01)	64.50 (63.63)
ain	GlobalMinimumAngle	146	31.44 (38.31)	81.67 (83.13)	-120.00 (-120.00)	-30.00 (-15.45)	30.00 (21.10)	60.00 (72.78)	210.00 (237.80)
ain	normalizedGlobalMinimum	146	0.20 (0.19)	0.21 (0.28)	0.00 (-1.55)	0.02 (0.02)	0.14 (0.14)	0.32 (0.33)	0.99 (1.13)
ain	innerMinimumAngle	146	177.33 (184.08)	33.04 (36.33)	120.00 (99.90)	150.00 (156.90)	180.00 (188.35)	210.00 (207.80)	240.00 (246.00)
ain	innerMinimumVal	146	12.94 (12.11)	21.22 (21.03)	0.00 (-3.49)	1.52 (1.03)	5.00 (3.68)	16.30 (15.32)	138.00 (134.53)
ain	normalizedinnerMinimumVal	146	0.44 (0.40)	0.31 (0.37)	0.00 (-1.53)	0.22 (0.16)	0.41 (0.38)	0.62 (0.60)	1.55 (1.64)
ain	Bandwidth$^{left}_{75\%}$	145	74.07 (56.33)	21.81 (32.39)	30.00 (16.10)	60.00 (38.30)	60.00 (48.55)	90.00 (62.88)	150.00 (268.50)

Continued on next page

Table 7 – *Continued from previous page*

		count	mean	std	min	25%	50%	75%	max
ain	ΔWidth^{left}	145	66.83 (50.50)	67.06 (64.97)	-90.00 (-113.30)	30.00 (9.15)	60.00 (61.90)	120.00 (95.22)	240.00 (261.20)
ain	Dip^{left}	145	9.30 (9.88)	11.55 (10.99)	0.00 (0.00)	2.67 (3.22)	5.94 (6.02)	11.00 (12.77)	98.00 (62.14)
ain	normalizedDip^{left}	145	0.55 (0.80)	0.50 (2.30)	0.00 (0.00)	0.21 (0.25)	0.42 (0.47)	0.75 (0.80)	3.40 (27.61)
ain	InnerWidth^{left}	145	57.52 (66.95)	32.88 (36.26)	0.00 (0.00)	30.00 (46.40)	60.00 (57.95)	90.00 (79.80)	180.00 (194.90)
ain	InnerBandwidth$^{left}_{75\,\%}$	145	36.00 (25.52)	14.83 (15.28)	0.00 (0.00)	30.00 (17.05)	30.00 (22.25)	30.00 (29.67)	90.00 (102.10)
ain	Kurtosis^{left}	145	-0.88 (-0.85)	0.68 (0.69)	-1.99 (-1.92)	-1.39 (-1.34)	-1.06 (-1.01)	-0.51 (-0.64)	1.95 (1.88)
ain	Maximum^{left}	145	22.33 (21.98)	26.84 (26.92)	0.50 (0.03)	6.40 (5.72)	12.22 (11.84)	28.00 (27.63)	141.25 (150.08)
ain	MaximumAngle^{left}	145	120.00 (117.13)	33.54 (36.59)	60.00 (45.00)	90.00 (93.88)	120.00 (117.15)	150.00 (143.05)	210.00 (190.40)
ain	normalizedMaximum^{left}	145	0.99 (1.20)	0.58 (2.16)	0.09 (0.01)	0.65 (0.66)	0.87 (0.91)	1.20 (1.25)	4.80 (26.07)
ain	OuterWidth^{left}	145	124.34 (117.45)	61.03 (52.19)	30.00 (21.90)	90.00 (70.85)	120.00 (116.40)	150.00 (148.25)	330.00 (338.60)
ain	OuterBandwidth$^{left}_{75\,\%}$	145	38.07 (30.81)	16.30 (27.36)	30.00 (8.10)	30.00 (19.02)	30.00 (24.05)	30.00 (33.60)	90.00 (265.70)
ain	PeakToPeak^{left}	145	16.25 (16.14)	19.11 (18.93)	0.50 (0.13)	4.90 (4.49)	10.00 (10.06)	21.10 (21.51)	135.00 (104.42)
ain	normalizedPeakToPeak^{left}	145	0.79 (1.01)	0.57 (2.30)	0.07 (0.05)	0.44 (0.44)	0.64 (0.69)	0.96 (1.03)	4.58 (27.62)
ain	Width^{left}	145	181.86 (184.40)	71.52 (62.10)	60.00 (60.40)	150.00 (144.53)	180.00 (180.60)	210.00 (220.02)	450.00 (416.00)
ain	maxAngleDist	145	124.97 (132.78)	42.28 (44.84)	30.00 (41.50)	90.00 (100.78)	120.00 (119.90)	150.00 (166.10)	240.00 (270.00)
ain	minusLeftSkewness	145	-0.19 (0.02)	0.63 (0.64)	-1.95 (-1.57)	-0.59 (-0.41)	-0.14 (0.04)	0.22 (0.50)	1.30 (1.57)
ain	outerMinimumAngle	146	128.84 (159.96)	134.26 (145.39)	0.00 (0.40)	30.00 (25.68)	60.00 (74.60)	300.00 (321.70)	330.00 (357.50)
ain	PeakToPeak	146	20.48 (20.34)	22.04 (22.60)	1.46 (0.33)	6.60 (5.29)	12.50 (12.58)	26.30 (26.61)	135.00 (119.88)
ain	normalizedPeakToPeak	146	1.05 (1.34)	0.72 (2.80)	0.25 (0.22)	0.62 (0.60)	0.85 (0.87)	1.30 (1.35)	4.58 (33.05)
ain	Bandwidth$^{right}_{75\,\%}$	146	81.78 (62.54)	33.34 (41.07)	30.00 (7.30)	60.00 (39.50)	60.00 (50.40)	90.00 (71.95)	270.00 (274.30)
ain	ΔWidth^{right}	146	62.88 (58.74)	71.63 (64.35)	-90.00 (-83.60)	0.00 (8.53)	60.00 (62.40)	120.00 (96.83)	240.00 (239.00)
ain	Dip^{right}	145	10.59 (11.05)	12.49 (13.63)	0.00 (0.00)	3.80 (3.32)	6.80 (6.56)	12.00 (13.65)	95.50 (104.98)
ain	normalizedDip^{right}	145	0.63 (0.94)	0.62 (2.80)	0.00 (0.00)	0.26 (0.28)	0.49 (0.53)	0.82 (0.85)	4.35 (33.04)
ain	InnerWidth^{right}	146	67.60 (65.83)	35.38 (31.11)	0.00 (0.00)	30.00 (48.65)	60.00 (61.55)	90.00 (75.90)	150.00 (141.60)
ain	InnerBandwidth$^{right}_{75\,\%}$	146	36.99 (25.71)	16.54 (15.61)	0.00 (0.00)	30.00 (17.65)	30.00 (22.10)	30.00 (31.48)	120.00 (85.20)
ain	Kurtosis^{right}	146	-0.87 (-0.83)	0.76 (0.81)	-1.92 (-1.93)	-1.46 (-1.40)	-1.02 (-0.99)	-0.67 (-0.56)	2.71 (2.76)
ain	Maximum^{right}	146	23.48 (23.16)	27.41 (28.07)	0.75 (0.45)	6.71 (5.29)	13.50 (12.80)	30.00 (30.68)	150.57 (151.46)
ain	MaximumAngle^{right}	146	244.93 (249.91)	32.87 (33.51)	150.00 (160.30)	240.00 (228.45)	240.00 (247.10)	270.00 (271.52)	300.00 (315.00)
ain	normalizedMaximum^{right}	146	1.08 (1.34)	0.66 (2.66)	0.21 (0.21)	0.68 (0.65)	0.90 (0.96)	1.30 (1.36)	4.53 (31.50)
ain	OuterWidth^{right}	146	130.48 (124.57)	61.39 (56.50)	30.00 (42.30)	90.00 (79.45)	120.00 (125.40)	172.50 (150.97)	300.00 (333.60)
ain	OuterBandwidth$^{right}_{75\,\%}$	146	44.79 (36.83)	30.15 (37.52)	30.00 (7.30)	30.00 (19.70)	30.00 (24.95)	60.00 (40.25)	240.00 (267.50)
ain	PeakToPeak^{right}	146	17.44 (17.32)	19.47 (20.74)	0.33 (0.33)	4.95 (4.36)	10.00 (9.96)	21.18 (21.35)	108.90 (119.88)
ain	normalizedPeakToPeak^{right}	146	0.87 (1.15)	0.65 (2.80)	0.12 (0.03)	0.49 (0.48)	0.70 (0.72)	1.08 (1.07)	4.53 (33.05)
ain	Skewness^{right}	146	0.23 (-0.01)	0.67 (0.69)	-1.36 (-1.83)	-0.25 (-0.42)	0.26 (0.01)	0.68 (0.43)	2.02 (1.59)
ain	Width^{right}	146	198.08 (190.40)	70.07 (64.65)	60.00 (43.60)	150.00 (145.80)	180.00 (183.60)	240.00 (235.32)	450.00 (435.50)
ain	tcSymmetryIndex	146	0.39 (6.79)	0.12 (2.53)	0.11 (0.93)	0.31 (5.10)	0.40 (6.78)	0.48 (8.42)	0.72 (15.02)
uni	CircularVariance	146	0.59 (0.57)	0.21 (0.21)	0.13 (0.10)	0.42 (0.41)	0.62 (0.59)	0.74 (0.73)	0.99 (0.98)
uni	GlobalMaximum	146	24.40 (23.88)	27.07 (27.18)	1.04 (0.89)	6.95 (6.90)	14.19 (15.01)	30.35 (29.16)	157.22 (156.52)
uni	GlobalMaximumAngle	146	233.42 (241.27)	35.84 (48.46)	0.00 (42.80)	240.00 (230.75)	240.00 (244.10)	240.00 (255.23)	240.00 (358.80)
uni	normalizedGlobalMaximum	146	1.00 (1.00)	0.00 (0.00)	1.00 (1.00)	1.00 (1.00)	1.00 (1.00)	1.00 (1.00)	1.00 (1.00)
uni	GlobalMinimum	146	5.08 (5.07)	8.24 (8.50)	0.00 (-10.71)	0.56 (0.52)	1.96 (1.86)	5.42 (5.55)	52.93 (50.08)
uni	GlobalMinimumAngle	146	58.56 (53.81)	72.92 (69.54)	-60.00 (-80.30)	0.00 (0.00)	60.00 (52.10)	120.00 (98.80)	210.00 (239.80)
uni	normalizedGlobalMinimum	146	0.20 (0.21)	0.16 (0.19)	0.00 (-0.46)	0.07 (0.05)	0.16 (0.17)	0.30 (0.34)	0.69 (0.70)
uni	PeakToPeak	146	19.32 (18.80)	22.83 (23.20)	0.74 (0.66)	5.35 (5.84)	11.50 (11.16)	25.37 (23.71)	150.56 (150.27)

Continued on next page

Table 7 – *Continued from previous page*

		count	mean	std	min	25%	50%	75%	max
uni	normalizedPeakToPeak	146	0.80 (0.79)	0.16 (0.19)	0.31 (0.30)	0.70 (0.66)	0.84 (0.83)	0.93 (0.95)	1.00 (1.46)
uni	$\text{Bandwidth}^{\text{right}}_{75\,\%}$	146	84.66 (68.48)	29.41 (36.56)	60.00 (18.60)	60.00 (45.03)	90.00 (60.90)	90.00 (80.15)	210.00 (199.40)
uni	$\Delta\text{Width}^{\text{right}}$	146	-2.88 (-21.02)	145.84 (134.70)	-240.00 (-262.00)	-120.00 (-100.95)	0.00 (-25.20)	120.00 (57.45)	300.00 (264.00)
uni	$\text{InnerWidth}^{\text{right}}$	146	181.44 (190.51)	72.92 (67.35)	30.00 (48.00)	120.00 (151.27)	180.00 (192.60)	240.00 (230.47)	300.00 (311.00)
uni	$\text{InnerBandwidth}^{\text{right}}_{75\,\%}$	146	40.68 (33.93)	17.17 (21.25)	30.00 (9.30)	30.00 (22.53)	30.00 (29.50)	60.00 (37.58)	120.00 (159.60)
uni	$\text{Kurtosis}^{\text{right}}$	146	-0.55 (-0.21)	0.96 (1.99)	-1.69 (-1.93)	-1.19 (-1.19)	-0.83 (-0.85)	-0.24 (-0.18)	4.06 (6.66)
uni	$\text{Maximum}^{\text{right}}$	146	24.39 (23.77)	27.07 (27.23)	1.04 (0.83)	6.95 (6.87)	14.19 (15.01)	30.35 (29.16)	157.22 (156.52)
uni	$\text{MaximumAngle}^{\text{right}}$	146	240.00 (244.32)	0.00 (21.77)	240.00 (180.00)	240.00 (232.03)	240.00 (244.35)	240.00 (254.88)	240.00 (300.00)
uni	$\text{normalizedMaximum}^{\text{right}}$	146	1.00 (0.98)	0.00 (0.07)	0.96 (0.41)	1.00 (1.00)	1.00 (1.00)	1.00 (1.00)	1.00 (1.00)
uni	$\text{OuterWidth}^{\text{right}}$	146	178.56 (169.49)	72.92 (67.35)	60.00 (49.00)	120.00 (129.53)	180.00 (167.40)	240.00 (208.73)	330.00 (312.00)
uni	$\text{OuterBandwidth}^{\text{right}}_{75\,\%}$	146	43.97 (34.54)	21.77 (23.09)	30.00 (6.30)	30.00 (22.07)	30.00 (28.45)	60.00 (38.03)	150.00 (157.00)
uni	$\text{PeakToPeak}^{\text{right}}$	146	19.31 (18.70)	22.83 (23.23)	0.74 (0.25)	5.35 (5.60)	11.50 (11.09)	25.37 (23.71)	150.56 (150.27)
uni	$\text{normalizedPeakToPeak}^{\text{right}}$	146	0.80 (0.78)	0.17 (0.19)	0.31 (0.14)	0.69 (0.65)	0.84 (0.83)	0.93 (0.93)	1.00 (1.34)
uni	$\text{Skewness}^{\text{right}}$	146	0.53 (0.61)	0.57 (0.81)	-1.00 (-1.15)	0.14 (0.04)	0.55 (0.51)	0.91 (0.94)	2.17 (2.80)
uni	$\text{Width}^{\text{right}}$	146	360.00 (360.00)	0.00 (0.00)	360.00 (360.00)	360.00 (360.00)	360.00 (360.00)	360.00 (360.00)	360.00 (360.00)
uni vs afix	ΔGlobalMinimum	145	-0.10 (0.14)	5.42 (4.72)	-19.30 (-8.55)	-1.89 (-1.60)	-0.39 (-0.65)	1.00 (0.19)	30.00 (17.11)
uni vs afix	normalizedΔGlobalMinimum	145	-0.03 (-0.06)	0.24 (0.18)	-0.43 (-0.51)	-0.15 (-0.15)	-0.02 (-0.08)	0.07 (0.01)	2.00 (0.48)
uni vs afix	ΔRightMaximum	144	-5.58 (-7.15)	14.20 (17.87)	-59.22 (-55.90)	-8.64 (-8.68)	-2.59 (-1.66)	1.22 (1.21)	41.92 (31.60)
uni vs afix	normalizedΔRightMaximum	144	-0.06 (-0.03)	0.76 (0.74)	-0.93 (-0.90)	-0.47 (-0.50)	-0.22 (-0.22)	0.12 (0.23)	6.07 (3.20)
uni vs ain	ΔGlobalMinimum	146	0.96 (1.53)	6.04 (6.52)	-14.83 (-6.99)	-1.12 (-1.08)	0.00 (-0.09)	0.89 (1.07)	41.39 (28.01)
uni vs ain	normalizedΔGlobalMinimum	146	0.00 (0.02)	0.16 (0.20)	-0.35 (-0.32)	-0.08 (-0.09)	0.00 (-0.01)	0.08 (0.12)	0.60 (0.61)
uni vs ain	ΔRightMaximum	146	-0.91 (0.23)	15.75 (16.33)	-49.44 (-33.66)	-6.11 (-6.67)	-0.71 (-0.22)	2.93 (4.81)	76.94 (58.96)
uni vs ain	normalizedΔRightMaximum	146	0.08 (0.13)	0.66 (0.72)	-0.79 (-0.63)	-0.32 (-0.29)	-0.10 (-0.03)	0.30 (0.37)	3.53 (3.85)
