# Supplementary material for: Model-Free Estimation of Tuning Curves and Their Attentional Modulation, Based on Sparse and Noisy Data
Source: PLoS One. 2016 Jan 19;11(1):e0146500. doi: 10.1371/journal.pone.0146500 (PMC4718600; doi:10.1371/journal.pone.0146500)
Supplement: S8 Table — List of all significantly different feature pairs when evaluated with the direct method, as well as the corresponding p-value of the Kruskal-Wallis test, and medians, means and cell counts. (PDF) [file pone.0146500.s012.pdf]

Supporting Table S8

uni2 Comparisons

spatially separate: uni2
None significant.

transparent: uni2
None significant.

uni2-afix Comparisons

spatially separate: uni2-afix comparisons

					p	median1	median2	mean1	mean2	n1	n2
x	uni2	GlobalMinimumAngle	afix	GlobalMinimumAngle	0.000	60.00	0.00	64.59	15.69	85	109
x	uni2	Bandwidth$^{right}_{75\%}$	afix	Bandwidth$^{right}_{75\%}$	0.037	90.00	90.00	94.24	84.22	85	109
x	uni2	ΔWidth^{right}	afix	ΔWidth^{right}	0.000	0.00	60.00	9.18	60.55	85	109
x	uni2	InnerWidth^{right}	afix	InnerWidth^{right}	0.000	180.00	60.00	175.41	65.78	85	109
x	uni2	OuterWidth^{right}	afix	OuterWidth^{right}	0.000	180.00	120.00	184.59	126.33	85	109
x	uni2	Width^{right}	afix	Width^{right}	0.000	360.00	210.00	360.00	192.11	85	109
y	uni2	GlobalMinimum	afix	GlobalMinimum	0.017	4.50	7.14	7.36	13.52	85	109
y	uni2	normalizedMaximum^{right}	afix	normalizedMaximum^{right}	0.000	1.00	0.94	1.00	0.91	85	85
y	uni2	normalizedPeakToPeak^{right}	afix	normalizedPeakToPeak^{right}	0.000	0.86	0.68	0.83	0.69	85	85
shape	uni2	Kurtosis^{right}	afix	Kurtosis^{right}	0.000	-0.74	-1.22	-0.52	-1.07	85	109
shape	uni2	Skewness^{right}	afix	Skewness^{right}	0.000	0.63	0.14	0.69	0.08	85	109

transparent: uni2-afix comparisons

					p	median1	median2	mean1	mean2	n1	n2
x	uni2	GlobalMinimumAngle	afix	GlobalMinimumAngle	0.015	60.00	30.00	58.56	38.90	146	145
x	uni2	Bandwidth$^{right}_{75\%}$	afix	Bandwidth$^{right}_{75\%}$	0.049	90.00	60.00	84.66	79.17	146	144
x	uni2	ΔWidth^{right}	afix	ΔWidth^{right}	0.000	0.00	60.00	-2.88	68.54	146	144
x	uni2	InnerWidth^{right}	afix	InnerWidth^{right}	0.000	180.00	60.00	181.44	63.96	146	144
x	uni2	InnerBandwidth$^{right}_{75\%}$	afix	InnerBandwidth$^{right}_{75\%}$	0.006	30.00	30.00	40.68	35.83	146	144
x	uni2	OuterWidth^{right}	afix	OuterWidth^{right}	0.000	180.00	120.00	178.56	132.50	146	144
x	uni2	Width^{right}	afix	Width^{right}	0.000	360.00	195.00	360.00	196.46	146	144
y	uni2	normalizedGlobalMaximum	afix	normalizedGlobalMaximum	0.039	1.00	0.93	1.00	1.14	146	145
y	uni2	normalizedGlobalMinimum	afix	normalizedGlobalMinimum	0.023	0.16	0.12	0.20	0.17	146	145
y	uni2	normalizedMaximum^{right}	afix	normalizedMaximum^{right}	0.000	1.00	0.78	1.00	0.94	146	144
y	uni2	normalizedPeakToPeak^{right}	afix	normalizedPeakToPeak^{right}	0.000	0.84	0.55	0.80	0.76	146	144
shape	uni2	Kurtosis^{right}	afix	Kurtosis^{right}	0.000	-0.83	-1.09	-0.55	-0.89	146	145
shape	uni2	Skewness^{right}	afix	Skewness^{right}	0.000	0.55	0.24	0.53	0.26	146	145

spatially separate vs transparent: uni2-afix comparisons

		p	median1	median2	mean1	mean2	n1	n2
y	normalizedΔRightMaximum	0.021	-0.06	-0.22	-0.09	-0.06	85	144

afix Comparisons

spatially separate: afix

					p	median1	median2	mean1	mean2	n1	n2
x	afix	InnerBandwidth$^{\text{left}}_{75\,\%}$	afix	OuterBandwidth$^{\text{right}}_{75\,\%}$	0.027	30.00	30.00	39.36	44.86	109	109
x	afix	OuterWidth^{left}	afix	OuterWidth^{right}	0.002	120.00	120.00	110.37	126.33	109	109
x	afix	Width^{left}	afix	Width^{right}	0.000	150.00	210.00	167.89	192.11	109	109
x	afix	InnerBandwidth$^{\text{right}}_{75\,\%}$	afix	OuterBandwidth$^{\text{right}}_{75\,\%}$	0.017	30.00	30.00	39.36	44.86	109	109
y	afix	Dip^{left}	afix	Dip^{right}	0.010	9.80	13.50	12.92	15.90	109	109
shape	afix	Kurtosis^{left}	afix	Kurtosis^{right}	0.034	-1.02	-1.22	-0.93	-1.07	109	109

transparent: afix

					p	median1	median2	mean1	mean2	n1	n2
x	afix	InnerBandwidth$^{\text{left}}_{75\,\%}$	afix	OuterBandwidth$^{\text{right}}_{75\,\%}$	0.007	30.00	30.00	36.04	43.33	144	144
x	afix	Width^{left}	afix	Width^{right}	0.014	180.00	195.00	186.25	196.46	144	144
x	afix	InnerBandwidth$^{\text{right}}_{75\,\%}$	afix	OuterBandwidth$^{\text{right}}_{75\,\%}$	0.003	30.00	30.00	35.83	43.33	144	144

spatially separate vs transparent: afix

		p	median1	median2	mean1	mean2	n1	n2
x	GlobalMinimumAngle	0.017	0.00	30.00	15.69	38.90	109	145
x	$\text{Bandwidth}^{\text{right}}_{75\,\%}$	0.032	90.00	60.00	84.22	79.17	109	144
y	ΔMaximum	0.012	3.60	0.00	2.98	0.97	109	143
y	ΔPeakToPeak	0.012	3.60	0.00	2.98	0.97	109	143
y	Dip	0.000	12.50	6.50	14.41	8.12	109	143
y	GlobalMaximum	0.000	34.50	14.00	43.41	21.15	109	145
y	GlobalMinimum	0.000	7.14	2.00	13.52	5.01	109	145
y	normalizedGlobalMinimum	0.006	0.20	0.12	0.22	0.17	85	145
y	innerMinimumVal	0.000	16.00	4.00	23.84	10.48	109	145
y	normalizedinnerMinimumVal	0.000	0.49	0.32	0.47	0.37	85	145
y	Dip^{left}	0.000	9.80	5.83	12.92	7.59	109	144
y	$\text{Maximum}^{\text{left}}$	0.000	29.50	11.00	36.76	18.14	109	144
y	$\text{PeakToPeak}^{\text{left}}$	0.000	21.00	9.08	23.24	13.09	109	144
y	PeakToPeak	0.000	26.80	11.93	29.89	16.14	109	145
y	$\text{Dip}^{\text{right}}$	0.000	13.50	6.07	15.90	8.60	109	143
y	$\text{Maximum}^{\text{right}}$	0.000	29.60	12.00	39.73	19.10	109	144
y	$\text{normalizedMaximum}^{\text{right}}$	0.021	0.94	0.78	0.91	0.94	85	144
y	$\text{PeakToPeak}^{\text{right}}$	0.000	22.90	9.50	26.22	14.05	109	144
shape	ΔSkewness	0.009	0.11	0.58	0.18	0.51	109	144

afix-ain Comparisons

spatially separate: afix-ain comparisons

					p	median1	median2	mean1	mean2	n1	n2
x	afix	ΔInnerWidth	ain	ΔInnerWidth	0.004	0.00	30.00	8.26	25.32	109	109
x	afix	$\text{Bandwidth}^{\text{left}}_{75\,\%}$	ain	$\text{Bandwidth}^{\text{left}}_{75\,\%}$	0.040	90.00	60.00	82.57	74.59	109	109
x	afix	$\text{InnerWidth}^{\text{left}}$	ain	$\text{InnerWidth}^{\text{left}}$	0.011	60.00	30.00	57.52	48.17	109	109
x	afix	$\text{InnerBandwidth}^{\text{left}}_{75\,\%}$	ain	$\text{InnerBandwidth}^{\text{left}}_{75\,\%}$	0.020	30.00	30.00	39.36	33.30	109	109
x	afix	$\text{Bandwidth}^{\text{right}}_{75\,\%}$	ain	$\text{Bandwidth}^{\text{right}}_{75\,\%}$	0.004	90.00	90.00	84.22	94.95	109	109
x	afix	$\text{InnerBandwidth}^{\text{right}}_{75\,\%}$	ain	$\text{InnerBandwidth}^{\text{right}}_{75\,\%}$	0.010	30.00	30.00	39.36	46.24	109	109
y	afix	ΔMaximum	ain	ΔMaximum	0.000	3.60	9.13	2.98	10.40	109	109
y	afix	normalizedΔMaximum	ain	normalizedΔMaximum	0.000	0.08	0.26	0.03	0.29	85	85
y	afix	ΔPeakToPeak	ain	ΔPeakToPeak	0.000	3.60	9.13	2.98	10.40	109	109
y	afix	normalizedΔPeakToPeak	ain	normalizedΔPeakToPeak	0.000	0.08	0.26	0.03	0.29	85	85
y	afix	Dip^{left}	ain	Dip^{left}	0.000	9.80	5.96	12.92	8.03	109	109
y	afix	$\text{normalizedPeakToPeak}^{\text{left}}$	ain	$\text{normalizedPeakToPeak}^{\text{left}}$	0.010	0.60	0.50	0.65	0.54	85	85
y	afix	$\text{normalizedMaximum}^{\text{right}}$	ain	$\text{normalizedMaximum}^{\text{right}}$	0.000	0.94	1.04	0.91	1.09	85	85
y	afix	$\text{normalizedPeakToPeak}^{\text{right}}$	ain	$\text{normalizedPeakToPeak}^{\text{right}}$	0.013	0.68	0.76	0.69	0.83	85	85
shape	afix	ΔSkewness	ain	ΔSkewness	0.044	0.11	-0.13	0.18	-0.04	109	109
shape	afix	$\text{Kurtosis}^{\text{left}}$	ain	$\text{Kurtosis}^{\text{left}}$	0.032	-1.02	-1.25	-0.93	-1.07	109	109
shape	afix	$\text{Skewness}^{\text{right}}$	ain	$\text{Skewness}^{\text{right}}$	0.006	0.14	-0.04	0.08	-0.06	109	109

transparent: afix-ain comparisons

					p	median1	median2	mean1	mean2	n1	n2
y	afix	normalizedGlobalMaximum	ain	normalizedGlobalMaximum	0.004	0.93	1.08	1.14	1.25	145	146
y	afix	normalizedinnerMinimumVal	ain	normalizedinnerMinimumVal	0.008	0.32	0.41	0.37	0.44	145	146
y	afix	$\text{normalizedMaximum}^{\text{left}}$	ain	$\text{normalizedMaximum}^{\text{left}}$	0.013	0.76	0.87	0.92	0.99	144	145
y	afix	$\text{normalizedPeakToPeak}^{\text{left}}$	ain	$\text{normalizedPeakToPeak}^{\text{left}}$	0.038	0.51	0.64	0.74	0.79	144	145
y	afix	normalizedPeakToPeak	ain	normalizedPeakToPeak	0.015	0.74	0.85	0.97	1.05	145	146
y	afix	$\text{normalizedMaximum}^{\text{right}}$	ain	$\text{normalizedMaximum}^{\text{right}}$	0.005	0.78	0.90	0.94	1.08	144	146
y	afix	$\text{normalizedPeakToPeak}^{\text{right}}$	ain	$\text{normalizedPeakToPeak}^{\text{right}}$	0.013	0.55	0.70	0.76	0.87	144	146

spatially separate vs transparent: afix-ain comparisons

		p	median1	median2	mean1	mean2	n1	n2
y	ΔLeftMaximum	0.000	-1.00	1.70	-1.05	4.35	109	143
y	normalizedΔLeftMaximum	0.000	-0.05	0.13	-0.08	0.08	85	143
y	ΔRightMaximum	0.018	4.57	1.65	6.37	4.65	109	144

ain Comparisons

spatially separate: ain

					p	median1	median2	mean1	mean2	n1	n2
x	ain	Bandwidth$^{left}_{75\,\%}$	ain	Bandwidth$^{right}_{75\,\%}$	0.000	60.00	90.00	74.59	94.95	109	109
x	ain	InnerWidth^{left}	ain	InnerWidth^{right}	0.000	30.00	90.00	48.17	73.49	109	109
x	ain	InnerBandwidth$^{left}_{75\,\%}$	ain	OuterBandwidth$^{left}_{75\,\%}$	0.002	30.00	30.00	33.30	41.28	109	109
x	ain	InnerBandwidth$^{left}_{75\,\%}$	ain	InnerBandwidth$^{right}_{75\,\%}$	0.000	30.00	30.00	33.30	46.24	109	109
x	ain	InnerBandwidth$^{left}_{75\,\%}$	ain	OuterBandwidth$^{right}_{75\,\%}$	0.000	30.00	30.00	33.30	48.72	109	109
x	ain	OuterWidth^{left}	ain	OuterWidth^{right}	0.017	120.00	120.00	114.77	126.88	109	109
x	ain	OuterBandwidth$^{left}_{75\,\%}$	ain	OuterBandwidth$^{right}_{75\,\%}$	0.013	30.00	30.00	41.28	48.72	109	109
x	ain	Width^{left}	ain	Width^{right}	0.000	150.00	210.00	162.94	200.37	109	109
y	ain	Dip^{left}	ain	Dip^{right}	0.000	5.96	15.45	8.03	18.43	109	109
y	ain	Maximum^{left}	ain	Maximum^{right}	0.003	27.11	36.29	35.71	46.10	109	109
y	ain	normalizedMaximum^{left}	ain	normalizedMaximum^{right}	0.000	0.79	1.04	0.80	1.09	85	85
y	ain	PeakToPeak^{left}	ain	PeakToPeak^{right}	0.000	17.14	26.60	20.24	30.64	109	109
y	ain	normalizedPeakToPeak^{left}	ain	normalizedPeakToPeak^{right}	0.000	0.50	0.76	0.54	0.83	85	85

transparent: ain

					p	median1	median2	mean1	mean2	n1	n2
x	ain	InnerWidth^{left}	ain	InnerWidth^{right}	0.014	60.00	60.00	57.52	67.60	145	146
x	ain	InnerBandwidth$^{left}_{75\,\%}$	ain	OuterBandwidth$^{right}_{75\,\%}$	0.019	30.00	30.00	36.00	44.79	145	146
x	ain	Width^{left}	ain	Width^{right}	0.013	180.00	180.00	181.86	198.08	145	146
x	ain	InnerBandwidth$^{right}_{75\,\%}$	ain	OuterBandwidth$^{right}_{75\,\%}$	0.017	30.00	30.00	36.99	44.79	146	146

spatially separate vs transparent: ain

		p	median1	median2	mean1	mean2	n1	n2
x	ΔInnerWidth	0.010	30.00	0.00	25.32	9.93	109	145
x	$\text{InnerWidth}^{\text{left}}$	0.029	30.00	60.00	48.17	57.52	109	145
x	$\text{Width}^{\text{left}}$	0.032	150.00	180.00	162.94	181.86	109	145
x	$\text{Bandwidth}^{\text{right}}_{75\,\%}$	0.000	90.00	60.00	94.95	81.78	109	146
x	$\text{InnerBandwidth}^{\text{right}}_{75\,\%}$	0.000	30.00	30.00	46.24	36.99	109	146
x	$\text{OuterBandwidth}^{\text{right}}_{75\,\%}$	0.010	30.00	30.00	48.72	44.79	109	146
y	ΔMaximum	0.000	9.13	0.50	10.40	1.29	109	145
y	normalizedΔMaximum	0.000	0.26	0.05	0.29	0.08	85	145
y	ΔPeakToPeak	0.000	9.13	0.50	10.40	1.29	109	145
y	normalizedΔPeakToPeak	0.000	0.26	0.05	0.29	0.08	85	145
y	Dip	0.000	11.20	6.90	13.23	9.95	109	145
y	normalizedDip	0.001	0.35	0.48	0.39	0.59	85	145
y	GlobalMaximum	0.000	38.50	15.75	47.58	26.52	109	146
y	GlobalMinimum	0.000	7.71	1.93	15.47	6.04	109	146
y	normalizedGlobalMinimum	0.009	0.23	0.14	0.26	0.20	85	146
y	innerMinimumVal	0.000	18.80	5.00	27.68	12.94	109	146
y	normalizedinnerMinimumVal	0.001	0.57	0.41	0.56	0.44	85	146
y	$\text{Maximum}^{\text{left}}$	0.000	27.11	12.22	35.71	22.33	109	145
y	$\text{normalizedMaximum}^{\text{left}}$	0.020	0.79	0.87	0.80	0.99	85	145
y	$\text{PeakToPeak}^{\text{left}}$	0.000	17.14	10.00	20.24	16.25	109	145
y	$\text{normalizedPeakToPeak}^{\text{left}}$	0.000	0.50	0.64	0.54	0.79	85	145
y	minusLeftSkewness	0.034	0.01	-0.14	-0.02	-0.19	109	145
y	PeakToPeak	0.000	29.33	12.50	32.11	20.48	109	146
y	$\text{Dip}^{\text{right}}$	0.000	15.45	6.80	18.43	10.59	109	145
y	$\text{Maximum}^{\text{right}}$	0.000	36.29	13.50	46.10	23.48	109	146
y	$\text{normalizedMaximum}^{\text{right}}$	0.034	1.04	0.90	1.09	1.08	85	146
y	$\text{PeakToPeak}^{\text{right}}$	0.000	26.60	10.00	30.64	17.44	109	146
shape	ΔSkewness	0.000	-0.13	0.42	-0.04	0.43	109	145
shape	$\text{Kurtosis}^{\text{left}}$	0.021	-1.25	-1.06	-1.07	-0.88	109	145
shape	$\text{Kurtosis}^{\text{right}}$	0.047	-1.15	-1.02	-1.07	-0.87	109	146
shape	$\text{Skewness}^{\text{right}}$	0.000	-0.04	0.26	-0.06	0.23	109	146
shape	tcSymmetryIndex	0.009	0.45	0.40	0.44	0.39	109	146
