# Supplementary material for: Model-Free Estimation of Tuning Curves and Their Attentional Modulation, Based on Sparse and Noisy Data
Source: PLoS One. 2016 Jan 19;11(1):e0146500. doi: 10.1371/journal.pone.0146500 (PMC4718600; doi:10.1371/journal.pone.0146500)
Supplement: S9 Table — List of all significantly different feature pairs when evaluated with the best model “bM”, as well as the corresponding p-value of the Kruskal-Wallis test, and medians, means and cell counts. (PDF) [file pone.0146500.s013.pdf]

Supporting Table S9

uni2 Comparisons

spatially separate: uni2
None significant.

transparent: uni2
None significant.

uni2-afix Comparisons

spatially separate: uni2-afix comparisons

					p	median1	median2	mean1	mean2	n1	n2
x	uni2	GlobalMinimumAngle	afix	GlobalMinimumAngle	0.000	67.30	12.80	63.41	21.42	85	109
x	uni2	$\text{Bandwidth}^{\text{right}}_{75\,\%}$	afix	$\text{Bandwidth}^{\text{right}}_{75\,\%}$	0.002	61.60	54.80	69.70	58.21	85	109
x	uni2	$\Delta\text{Width}^{\text{right}}$	afix	$\Delta\text{Width}^{\text{right}}$	0.000	0.00	68.00	1.20	67.00	85	109
x	uni2	$\text{InnerWidth}^{\text{right}}$	afix	$\text{InnerWidth}^{\text{right}}$	0.000	180.00	61.70	179.40	63.12	85	109
x	uni2	$\text{InnerBandwidth}^{\text{right}}_{75\,\%}$	afix	$\text{InnerBandwidth}^{\text{right}}_{75\,\%}$	0.000	30.20	25.90	34.48	26.07	85	109
x	uni2	$\text{OuterWidth}^{\text{right}}$	afix	$\text{OuterWidth}^{\text{right}}$	0.000	180.00	133.90	180.60	130.12	85	109
x	uni2	$\text{Width}^{\text{right}}$	afix	$\text{Width}^{\text{right}}$	0.000	360.00	199.80	360.00	193.24	85	109
y	uni2	normalizedGlobalMaximum	afix	normalizedGlobalMaximum	0.003	1.00	1.08	1.00	1.10	85	85
y	uni2	GlobalMinimum	afix	GlobalMinimum	0.015	4.14	6.78	7.25	13.40	85	109
y	uni2	$\text{normalizedPeakToPeak}^{\text{right}}$	afix	$\text{normalizedPeakToPeak}^{\text{right}}$	0.001	0.87	0.74	0.83	0.73	85	85
shape	uni2	$\text{Kurtosis}^{\text{right}}$	afix	$\text{Kurtosis}^{\text{right}}$	0.000	-0.75	-1.21	-0.48	-0.98	85	109
shape	uni2	$\text{Skewness}^{\text{right}}$	afix	$\text{Skewness}^{\text{right}}$	0.000	0.64	0.06	0.71	0.02	85	109

transparent: uni2-afix comparisons

					p	median1	median2	mean1	mean2	n1	n2
x	uni2	$\text{Bandwidth}^{\text{right}}_{75\,\%}$	afix	$\text{Bandwidth}^{\text{right}}_{75\,\%}$	0.012	60.90	50.00	68.48	59.65	146	143
x	uni2	$\Delta\text{Width}^{\text{right}}$	afix	$\Delta\text{Width}^{\text{right}}$	0.000	-25.20	45.70	-21.02	46.73	146	143
x	uni2	$\text{InnerWidth}^{\text{right}}$	afix	$\text{InnerWidth}^{\text{right}}$	0.000	192.60	61.60	190.51	71.58	146	143
x	uni2	$\text{InnerBandwidth}^{\text{right}}_{75\,\%}$	afix	$\text{InnerBandwidth}^{\text{right}}_{75\,\%}$	0.000	29.50	23.00	33.93	27.69	146	143
x	uni2	$\text{OuterWidth}^{\text{right}}$	afix	$\text{OuterWidth}^{\text{right}}$	0.000	167.40	118.00	169.49	118.31	146	143
x	uni2	$\text{OuterBandwidth}^{\text{right}}_{75\,\%}$	afix	$\text{OuterBandwidth}^{\text{right}}_{75\,\%}$	0.048	28.45	25.60	34.54	31.96	146	143
x	uni2	$\text{Width}^{\text{right}}$	afix	$\text{Width}^{\text{right}}$	0.000	360.00	187.80	360.00	189.89	146	143
y	uni2	normalizedGlobalMaximum	afix	normalizedGlobalMaximum	0.003	1.00	0.89	1.00	1.69	146	143
y	uni2	$\text{normalizedMaximum}^{\text{right}}$	afix	$\text{normalizedMaximum}^{\text{right}}$	0.000	1.00	0.76	0.98	1.33	146	143
y	uni2	$\text{normalizedPeakToPeak}^{\text{right}}$	afix	$\text{normalizedPeakToPeak}^{\text{right}}$	0.003	0.83	0.62	0.78	0.93	146	143
shape	uni2	$\text{Kurtosis}^{\text{right}}$	afix	$\text{Kurtosis}^{\text{right}}$	0.020	-0.85	-0.98	-0.21	-0.74	146	143
shape	uni2	$\text{Skewness}^{\text{right}}$	afix	$\text{Skewness}^{\text{right}}$	0.000	0.51	-0.05	0.61	-0.03	146	143

spatially separate vs transparent: uni2-afix comparisons

		p	median1	median2	mean1	mean2	n1	n2
y	ΔRightMaximum	0.042	-0.24	-1.66	0.35	-7.15	27	40

afix Comparisons

spatially separate: afix

					p	median1	median2	mean1	mean2	n1	n2
x	afix	ΔOuterWidth	afix	ΔInnerWidth	0.004	32.60	8.70	22.18	4.31	109	109
x	afix	$\Delta\text{Width}^{\text{left}}$	afix	$\Delta\text{Width}^{\text{right}}$	0.002	50.60	68.00	49.12	67.00	109	109
x	afix	$\text{InnerWidth}^{\text{left}}$	afix	$\text{InnerWidth}^{\text{right}}$	0.042	56.30	61.70	58.82	63.12	109	109
x	afix	$\text{InnerBandwidth}^{\text{left}}_{75\,\%}$	afix	$\text{OuterBandwidth}^{\text{right}}_{75\,\%}$	0.017	25.50	28.80	27.89	32.14	109	109
x	afix	$\text{OuterWidth}^{\text{left}}$	afix	$\text{OuterWidth}^{\text{right}}$	0.000	106.70	133.90	107.94	130.12	109	109
x	afix	$\text{Width}^{\text{left}}$	afix	$\text{Width}^{\text{right}}$	0.000	160.20	199.80	166.76	193.24	109	109
x	afix	$\text{InnerBandwidth}^{\text{right}}_{75\,\%}$	afix	$\text{OuterBandwidth}^{\text{right}}_{75\,\%}$	0.016	25.90	28.80	26.07	32.14	109	109
y	afix	Dip^{left}	afix	$\text{Dip}^{\text{right}}$	0.037	11.25	14.56	14.46	17.13	109	109

transparent: afix

					p	median1	median2	mean1	mean2	n1	n2
x	afix	$\text{InnerBandwidth}^{\text{left}}_{75\,\%}$	afix	$\text{OuterBandwidth}^{\text{left}}_{75\,\%}$	0.036	22.40	23.60	25.20	32.24	143	143
x	afix	$\text{InnerBandwidth}^{\text{left}}_{75\,\%}$	afix	$\text{OuterBandwidth}^{\text{right}}_{75\,\%}$	0.013	22.40	25.60	25.20	31.96	143	143

spatially separate vs transparent: afix

		p	median1	median2	mean1	mean2	n1	n2
x	maxAngleDist	0.016	117.90	129.40	121.94	138.27	109	143
x	ΔWidth^{right}	0.004	68.00	45.70	67.00	46.73	109	143
x	MaximumAngle^{right}	0.005	240.20	247.50	238.85	251.45	109	143
x	OuterWidth^{right}	0.005	133.90	118.00	130.12	118.31	109	143
y	Dip	0.000	13.62	6.52	15.79	8.15	109	143
y	GlobalMaximum	0.000	35.15	12.94	44.06	20.66	109	143
y	normalizedGlobalMaximum	0.006	1.08	0.89	1.10	1.69	85	143
y	GlobalMinimum	0.000	6.78	1.53	13.40	5.08	109	143
y	normalizedGlobalMinimum	0.023	0.20	0.12	0.22	0.40	85	143
y	innerMinimumVal	0.000	15.29	4.12	22.79	9.94	109	143
y	normalizedinnerMinimumVal	0.001	0.46	0.32	0.46	0.69	85	143
y	Dip^{left}	0.000	11.25	5.10	14.46	7.36	109	143
y	Maximum^{left}	0.000	27.77	10.37	37.25	17.30	109	143
y	PeakToPeak^{left}	0.000	20.89	9.12	23.85	12.22	109	143
y	PeakToPeak	0.000	26.89	10.43	30.67	15.58	109	143
y	normalizedPeakToPeak	0.035	0.84	0.72	0.88	1.29	85	143
y	Dip^{right}	0.000	14.56	6.59	17.13	8.94	109	143
y	Maximum^{right}	0.000	30.34	11.57	39.92	18.88	109	143
y	normalizedMaximum^{right}	0.021	0.97	0.76	0.95	1.33	85	143
y	PeakToPeak^{right}	0.000	23.58	8.70	26.52	13.79	109	143
shape	Kurtosis^{right}	0.005	-1.21	-0.98	-0.98	-0.74	109	143

afix-ain Comparisons

spatially separate: afix-ain comparisons

					p	median1	median2	mean1	mean2	n1	n2
x	afix	ΔInnerWidth	ain	ΔInnerWidth	0.000	8.70	23.80	4.31	32.40	109	109
x	afix	innerMinimumAngle	ain	innerMinimumAngle	0.007	174.00	165.50	175.73	164.07	109	109
x	afix	InnerWidth^{left}	ain	InnerWidth^{left}	0.002	56.30	46.60	58.82	46.88	109	109
x	afix	Bandwidth$^{\text{right}}_{75\,\%}$	ain	Bandwidth$^{\text{right}}_{75\,\%}$	0.000	54.80	70.20	58.21	72.73	109	109
x	afix	ΔWidth^{right}	ain	ΔWidth^{right}	0.014	68.00	58.10	67.00	50.62	109	109
x	afix	InnerWidth^{right}	ain	InnerWidth^{right}	0.001	61.70	72.70	63.12	79.28	109	109
x	afix	InnerBandwidth$^{\text{right}}_{75\,\%}$	ain	InnerBandwidth$^{\text{right}}_{75\,\%}$	0.000	25.90	30.40	26.07	33.98	109	109
x	afix	OuterBandwidth$^{\text{right}}_{75\,\%}$	ain	OuterBandwidth$^{\text{right}}_{75\,\%}$	0.003	28.80	32.80	32.14	38.75	109	109
x	afix	Width^{right}	ain	Width^{right}	0.020	199.80	210.50	193.24	209.18	109	109
y	afix	ΔMaximum	ain	ΔMaximum	0.000	3.21	8.46	2.68	9.91	109	109
y	afix	normalizedΔMaximum	ain	normalizedΔMaximum	0.001	0.07	0.26	0.04	0.27	85	85
y	afix	ΔPeakToPeak	ain	ΔPeakToPeak	0.000	3.21	8.46	2.68	9.91	109	109
y	afix	normalizedΔPeakToPeak	ain	normalizedΔPeakToPeak	0.001	0.07	0.26	0.04	0.27	85	85
y	afix	Dip^{left}	ain	Dip^{left}	0.000	11.25	6.21	14.46	9.47	109	109
y	afix	PeakToPeak^{left}	ain	PeakToPeak^{left}	0.044	20.89	16.69	23.85	20.18	109	109
y	afix	normalizedPeakToPeak^{left}	ain	normalizedPeakToPeak^{left}	0.006	0.64	0.52	0.69	0.57	85	85
y	afix	normalizedMaximum^{right}	ain	normalizedMaximum^{right}	0.001	0.97	1.07	0.95	1.11	85	85
shape	afix	Skewness^{right}	ain	Skewness^{right}	0.049	0.06	-0.12	0.02	-0.07	109	109
shape	afix	tcSymmetryIndex	ain	tcSymmetryIndex	0.050	7.08	8.12	6.86	7.58	109	109

transparent: afix-ain comparisons

					p	median1	median2	mean1	mean2	n1	n2
y	afix	normalizedGlobalMaximum	ain	normalizedGlobalMaximum	0.002	0.89	1.14	1.69	1.53	143	146
y	afix	normalizedMaximum^{left}	ain	normalizedMaximum^{left}	0.004	0.75	0.91	1.49	1.20	143	146
y	afix	normalizedPeakToPeak^{left}	ain	normalizedPeakToPeak^{left}	0.011	0.53	0.69	1.09	1.01	143	146
y	afix	normalizedPeakToPeak	ain	normalizedPeakToPeak	0.011	0.72	0.87	1.29	1.34	143	146
y	afix	normalizedMaximum^{right}	ain	normalizedMaximum^{right}	0.023	0.76	0.96	1.33	1.34	143	146

spatially separate vs transparent: afix-ain comparisons

		p	median1	median2	mean1	mean2	n1	n2
y	ΔLeftMaximum	0.000	-3.10	3.11	-2.16	8.18	36	39
y	normalizedΔLeftMaximum	0.000	-0.17	0.20	-0.14	0.17	31	39

ain Comparisons

spatially separate: ain

					p	median1	median2	mean1	mean2	n1	n2
x	ain	$\textsc{Bandwidth}^{\text{left}}_{75\,\%}$	ain	$\textsc{Bandwidth}^{\text{right}}_{75\,\%}$	0.000	50.50	70.20	57.65	72.73	109	109
x	ain	$\textsc{InnerWidth}^{\text{left}}$	ain	$\textsc{InnerWidth}^{\text{right}}$	0.000	46.60	72.70	46.88	79.28	109	109
x	ain	$\textsc{InnerBandwidth}^{\text{left}}_{75\,\%}$	ain	$\textsc{OuterBandwidth}^{\text{left}}_{75\,\%}$	0.007	23.40	26.90	23.75	33.90	109	109
x	ain	$\textsc{InnerBandwidth}^{\text{left}}_{75\,\%}$	ain	$\textsc{InnerBandwidth}^{\text{right}}_{75\,\%}$	0.000	23.40	30.40	23.75	33.98	109	109
x	ain	$\textsc{InnerBandwidth}^{\text{left}}_{75\,\%}$	ain	$\textsc{OuterBandwidth}^{\text{right}}_{75\,\%}$	0.000	23.40	32.80	23.75	38.75	109	109
x	ain	$\textsc{OuterWidth}^{\text{left}}$	ain	$\textsc{OuterWidth}^{\text{right}}$	0.000	103.40	133.40	110.55	129.90	109	109
x	ain	$\textsc{OuterBandwidth}^{\text{left}}_{75\,\%}$	ain	$\textsc{InnerBandwidth}^{\text{right}}_{75\,\%}$	0.028	26.90	30.40	33.90	33.98	109	109
x	ain	$\textsc{OuterBandwidth}^{\text{left}}_{75\,\%}$	ain	$\textsc{OuterBandwidth}^{\text{right}}_{75\,\%}$	0.000	26.90	32.80	33.90	38.75	109	109
x	ain	$\textsc{Width}^{\text{left}}$	ain	$\textsc{Width}^{\text{right}}$	0.000	150.60	210.50	157.42	209.18	109	109
y	ain	$\textsc{Dip}^{\text{left}}$	ain	$\textsc{Dip}^{\text{right}}$	0.000	6.21	15.64	9.47	19.38	109	109
y	ain	$\textsc{Maximum}^{\text{left}}$	ain	$\textsc{Maximum}^{\text{right}}$	0.005	27.89	35.76	35.65	45.56	109	109
y	ain	$\textsc{normalizedMaximum}^{\text{left}}$	ain	$\textsc{normalizedMaximum}^{\text{right}}$	0.000	0.84	1.07	0.83	1.11	85	85
y	ain	$\textsc{PeakToPeak}^{\text{left}}$	ain	$\textsc{PeakToPeak}^{\text{right}}$	0.000	16.69	25.40	20.18	30.09	109	109
y	ain	$\textsc{normalizedPeakToPeak}^{\text{left}}$	ain	$\textsc{normalizedPeakToPeak}^{\text{right}}$	0.000	0.52	0.81	0.57	0.84	85	85

transparent: ain

					p	median1	median2	mean1	mean2	n1	n2
x	ain	$\textsc{InnerBandwidth}^{\text{left}}_{75\,\%}$	ain	$\textsc{OuterBandwidth}^{\text{right}}_{75\,\%}$	0.004	22.25	24.95	25.52	36.83	146	146
x	ain	$\textsc{InnerBandwidth}^{\text{right}}_{75\,\%}$	ain	$\textsc{OuterBandwidth}^{\text{right}}_{75\,\%}$	0.003	22.10	24.95	25.71	36.83	146	146

spatially separate vs transparent: ain

		p	median1	median2	mean1	mean2	n1	n2
x	ΔInnerWidth	0.000	23.80	3.45	32.40	-1.12	109	146
x	ΔWidth	0.002	49.40	1.10	31.94	-3.86	109	146
x	innerMinimumAngle	0.000	165.50	188.35	164.07	184.08	109	146
x	$\text{InnerWidth}^{\text{left}}$	0.000	46.60	57.95	46.88	66.95	109	146
x	$\text{Width}^{\text{left}}$	0.000	150.60	180.60	157.42	184.40	109	146
x	$\text{Bandwidth}^{\text{right}}_{75\,\%}$	0.000	70.20	50.40	72.73	62.54	109	146
x	$\text{InnerWidth}^{\text{right}}$	0.002	72.70	61.55	79.28	65.83	109	146
x	$\text{InnerBandwidth}^{\text{right}}_{75\,\%}$	0.000	30.40	22.10	33.98	25.71	109	146
x	$\text{OuterBandwidth}^{\text{right}}_{75\,\%}$	0.000	32.80	24.95	38.75	36.83	109	146
x	$\text{Width}^{\text{right}}$	0.001	210.50	183.60	209.18	190.40	109	146
y	ΔMaximum	0.000	8.46	0.40	9.91	1.18	109	146
y	normalizedΔMaximum	0.000	0.26	0.05	0.27	0.14	85	146
y	ΔPeakToPeak	0.000	8.46	0.40	9.91	1.18	109	146
y	normalizedΔPeakToPeak	0.000	0.26	0.05	0.27	0.14	85	146
y	Dip	0.000	12.15	7.01	14.43	10.46	109	146
y	normalizedDip	0.000	0.38	0.57	0.42	0.87	85	146
y	GlobalMaximum	0.000	37.87	15.93	47.18	26.18	109	146
y	GlobalMinimum	0.000	8.09	1.44	15.47	5.84	109	146
y	normalizedGlobalMinimum	0.007	0.23	0.14	0.27	0.19	85	146
y	innerMinimumVal	0.000	17.35	3.68	26.18	12.11	109	146
y	normalizedinnerMinimumVal	0.001	0.51	0.38	0.55	0.40	85	146
y	$\text{Maximum}^{\text{left}}$	0.000	27.89	11.84	35.65	21.98	109	146
y	$\text{normalizedMaximum}^{\text{left}}$	0.041	0.84	0.91	0.83	1.20	85	146
y	$\text{PeakToPeak}^{\text{left}}$	0.001	16.69	10.06	20.18	16.14	109	146
y	$\text{normalizedPeakToPeak}^{\text{left}}$	0.000	0.52	0.69	0.57	1.01	85	146
y	PeakToPeak	0.000	28.10	12.58	31.71	20.34	109	146
y	$\text{Dip}^{\text{right}}$	0.000	15.64	6.56	19.38	11.05	109	146
y	$\text{Maximum}^{\text{right}}$	0.000	35.76	12.80	45.56	23.16	109	146
y	$\text{PeakToPeak}^{\text{right}}$	0.000	25.40	9.96	30.09	17.32	109	146
shape	$\text{Kurtosis}^{\text{left}}$	0.031	-1.18	-1.01	-0.98	-0.85	109	146
shape	$\text{Kurtosis}^{\text{right}}$	0.029	-1.21	-0.99	-1.10	-0.83	109	146
shape	tcSymmetryIndex	0.016	8.12	6.78	7.58	6.79	109	146
