# Supplementary material for: Model-Free Estimation of Tuning Curves and Their Attentional Modulation, Based on Sparse and Noisy Data
Source: PLoS One. 2016 Jan 19;11(1):e0146500. doi: 10.1371/journal.pone.0146500 (PMC4718600; doi:10.1371/journal.pone.0146500)
Supplement: S10 Table — Model functions and their parameters are described in Methods subsection “Model functions”, number before model indicates if the model pertained to data from the one or two stimulus conditions. u = mini yi+1.2ptpi yi where ptpi yi = maxi yi − mini yi and {yi} is the set of all firing rates in the tuning curve. The values of 10.74, 2.41 and 100 for the width-parameter k of the sβ model correspond to a half-width-at-half-maximum of 45°, 90°and 15°. Likewise the values of k = 2.3, 0 and 20.34 for the vM model correspond to 45°, 90°and 15°; As k = 0 would make the denominator in the definition of vM zero it was replaced by 0.001. (PDF) [file pone.0146500.s014.pdf]

Supporting Table S 10: Initial conditions and bounds for least-squares-fits. Model functions and their parameters are described in Methods subsection "Model functions", number before model indicates if the model pertained to data from the one or two stimulus conditions. $u = \min_i y_i + 1.2\,\mathrm{ptp}_i\, y_i$ where $\mathrm{ptp}_i\, y_i = \max_i y_i - \min_i y_i$ and $\{y_i\}$ is the set of all firing rates in the tuning curve. The values of 10.74, 2.41 and 100 for the width-parameter k of the sβ model correspond to a half-width-at-half-maximum of 45°, 90°and 15°. Likewise the values of $k = 2.3$, 0 and 20.34 for the vM model correspond to 45°, 90°and 15°; As $k = 0$ would make the denominator in the definition of vM zero it was replaced by 0.001.

model	initial condition	lower bounds	upper bounds
1 sβ	$(u/2, 10.74, 240, 10)$	$(0, 2.41, 180, -50)$	$(u, 100, 300, 100)$
1 vM	$(u/2, 2.3, 240, 10)$	$(0, 0.001, 180, -50)$	$(u, 20.34, 300, 100)$
1 wG	$(u/2, 45, 240, 10)$	$(0, 15, 180, -50)$	$(u, 90, 300, 100)$
1 wC	$(u/2, 45, 240, 10)$	$(0, 15, 180, -50)$	$(u, 90, 300, 100)$
1 wB	$(u/2, 45, 240, 10, 2)$	$(0, 15, 180, -50, 1)$	$(u, 90, 300, 100, 10)$
2 sβ	$(u/2, 10.74, 120, 5, u/2, 10.74, 240, 5)$	$(0, 2.41, 30, -25, 0, 2.41, 180, -25)$	$(u, 100, 180, 50, u, 100, 330, 50)$
2 vM	$(u/2, 2.3, 120, 5, u/2, 2.3, 240, 5)$	$(0, 0.001, 30, -25, 0, 0.001, 180, -25)$	$(u, 20.34, 180, 50, u, 20.34, 330, 50)$
2 wG	$(u/2, 45, 120, 5, u/2, 45, 240, 5)$	$(0, 15, 30, -25, 0, 15, 180, -25)$	$(u, 90, 180, 50, u, 90, 330, 50)$
2 wC	$(u/2, 45, 120, 5, u/2, 45, 240, 5)$	$(0, 15, 30, -25, 0, 15, 180, -25)$	$(u, 90, 180, 50, u, 90, 330, 50)$
2 wB	$(u/2, 45, 120, 5, 2, u/2, 45, 240, 5, 2)$	$(0, 15, 30, -25, 1, 0, 15, 180, -25, 1)$	$(u, 90, 180, 50, 10, u, 90, 330, 50, 10)$
